# Cohort Profile: Post-Hospitalisation COVID-19 (PHOSP-COVID) study

**DOI:** 10.1093/ije/dyad165

**Published:** 2023-12-18

**Authors:** Omer Elneima, Hamish J C McAuley, Olivia C Leavy, James D Chalmers, Alex Horsley, Ling-Pei Ho, Michael Marks, Krisnah Poinasamy, Betty Raman, Aarti Shikotra, Amisha Singapuri, Marco Sereno, Victoria C Harris, Linzy Houchen-Wolloff, Ruth M Saunders, Neil J Greening, Matthew Richardson, Jennifer K Quint, Andrew Briggs, Annemarie B Docherty, Steven Kerr, Ewen M Harrison, Nazir I Lone, Mathew Thorpe, Liam G Heaney, Keir E Lewis, Raminder Aul, Paul Beirne, Charlotte E Bolton, Jeremy S Brown, Gourab Choudhury, Nawar Diar Bakerly, Nicholas Easom, Carlos Echevarria, Jonathan Fuld, Nick Hart, John R Hurst, Mark G Jones, Dhruv Parekh, Paul Pfeffer, Najib M Rahman, Sarah L Rowland-Jones, Aa Roger Thompson, Caroline Jolley, Ajay M Shah, Dan G Wootton, Trudie Chalder, Melanie J Davies, Anthony De Soyza, John R Geddes, William Greenhalf, Simon Heller, Luke S Howard, Joseph Jacob, R Gisli Jenkins, Janet M Lord, William D-C Man, Gerry P McCann, Stefan Neubauer, Peter Jm Openshaw, Joanna C Porter, Matthew J Rowland, Janet T Scott, Malcolm G Semple, Sally J Singh, David C Thomas, Mark Toshner, Nikki Smith, Aziz Sheikh, Christopher E Brightling, Louise V Wain, Rachael A Evans, PHOSP-COVID Collaborative Group, C E Brightling, R A Evans, L V Wain, J D Chalmers, V C Harris, L P Ho, A Horsley, M Marks, K Poinasamy, B Raman, A Shikotra, A Singapuri, C E Brightling, R A Evans, L V Wain, R Dowling, C Edwardson, O Elneima, S Finney, N J Greening, B Hargadon, V C Harris, L Houchen-Wolloff, O C Leavy, H J C McAuley, C Overton, T Plekhanova, R M Saunders, M Sereno, A Singapuri, A Shikotra, C Taylor, S Terry, C Tong, B Zhao, D Lomas, E Sapey, C Berry, C E Bolton, N Brunskill, E R Chilvers, R Djukanovic, Y Ellis, D Forton, N French, J George, N A Hanley, N Hart, L McGarvey, N Maskell, H McShane, M Parkes, D Peckham, P Pfeffer, A Sayer, A Sheikh, A A R Thompson, N Williams, C E Brightling, W Greenhalf, M G Semple, M Ashworth, H E Hardwick, L Lavelle-Langham, W Reynolds, M Sereno, R M Saunders, A Singapuri, V Shaw, A Shikotra, B Venson, L V Wain, A B Docherty, E M Harrison, A Sheikh, J K Baillie, C E Brightling, L Daines, R Free, R A Evans, S Kerr, O C Leavy, N I Lone, H J C McAuley, R Pius, J K Quint, M Richardson, M Sereno, M Thorpe, L V Wain, M Halling-Brown, F Gleeson, J Jacob, S Neubauer, B Raman, S Siddiqui, J M Wild, S Aslani, G Baxter, M Beggs, C Bloomfield, M P Cassar, A Chiribiri, E Cox, D J Cuthbertson, M Halling-Brown, V M Ferreira, L Finnigan, S Francis, P Jezzard, G J Kemp, H Lamlum, E Lukaschuk, C Manisty, G P McCann, C McCracken, K McGlynn, R Menke, C A Miller, A J Moss, T E Nichols, C Nikolaidou, C O'Brien, G Ogbole, B Rangelov, D P O'Regan, A Pakzad, S Piechnik, S Plein, I Propescu, A A Samat, L Saunders, Z B Sanders, R Steeds, T Treibel, E M Tunnicliffe, M Webster, J Willoughby, J Weir McCall, C Xie, M Xu, L V Wain, J K Baillie, H Baxendale, C E Brightling, M Brown, J D Chalmers, R A Evans, B Gooptu, W Greenhalf, H E Hardwick, R G Jenkins, D Jones, I Koychev, C Langenberg, A Lawrie, P L Molyneaux, A Shikotra, J Pearl, M Ralser, N Sattar, R M Saunders, J T Scott, T Shaw, D Thomas, D Wilkinson, L G Heaney, A De Soyza, D Adeloye, C E Brightling, J S Brown, J Busby, J D Chalmers, C Echevarria, L Daines, O Elneima, R A Evans, J R Hurst, P Novotny, C Nicolaou, P Pfeffer, K Poinasamy, J K Quint, I Rudan, E Sapey, M Shankar-Hari, A Sheikh, S Siddiqui, S Walker, B Zheng, J R Geddes, M Hotopf, K Abel, R Ahmed, L Allan, C Armour, D Baguley, D Baldwin, C Ballard, K Bhui, G Breen, K Breeze, M Broome, T Brugha, E Bullmore, D Burn, F Callard, J Cavanagh, T Chalder, D Clark, A David, B Deakin, H Dobson, B Elliott, J Evans, R A Evans, R Francis, E Guthrie, P Harrison, M Henderson, A Hosseini, N Huneke, M Husain, T Jackson, I Jones, T Kabir, P Kitterick, A Korszun, I Koychev, J Kwan, A Lingford-Hughes, P Mansoori, H McAllister-Williams, K McIvor, B Michael, L Milligan, R Morriss, E Mukaetova-Ladinska, K Munro, A Nevado-Holgado, T Nicholson, C Nicolaou, S Paddick, C Pariante, J Pimm, K Saunders, M Sharpe, G Simons, J P Taylor, R Upthegrove, S Wessely, G P McCann, S Amoils, C Antoniades, A Banerjee, A Bularga, C Berry, P Chowienczyk, J P Greenwood, A D Hughes, K Khunti, C Lawson, N L Mills, A J Moss, S Neubauer, B Raman, A N Sattar, C L Sudlow, M Toshner, P J M Openshaw, D Altmann, J K Baillie, R Batterham, H Baxendale, N Bishop, C E Brightling, P C Calder, R A Evans, J L Heeney, T Hussell, P Klenerman, J M Lord, P Moss, S L Rowland-Jones, W Schwaeble, M G Semple, R S Thwaites, L Turtle, L V Wain, S Walmsley, D Wraith, M J Rowland, A Rostron, J K Baillie, B Connolly, A B Docherty, N I Lone, D F McAuley, D Parekh, A Rostron, J Simpson, C Summers, R G Jenkins, J Porter, R J Allen, R Aul, J K Baillie, S Barratt, P Beirne, J Blaikley, R C Chambers, N Chaudhuri, C Coleman, E Denneny, L Fabbri, P M George, M Gibbons, F Gleeson, B Gooptu, B Guillen Guio, I Hall, N A Hanley, L P Ho, E Hufton, J Jacob, I Jarrold, G Jenkins, S Johnson, M G Jones, S Jones, F Khan, P Mehta, J Mitchell, P L Molyneaux, J E Pearl, K Piper Hanley, K Poinasamy, J Quint, D Parekh, P Rivera-Ortega, L C Saunders, M G Semple, J Simpson, D Smith, M Spears, L G Spencer, S Stanel, I Stewart, A A R Thompson, D Thickett, R Thwaites, L V Wain, S Walker, S Walsh, J M Wild, D G Wootton, L Wright, S Heller, M J Davies, H Atkins, S Bain, J Dennis, K Ismail, D Johnston, P Kar, K Khunti, C Langenberg, P McArdle, A McGovern, T Peto, J Petrie, E Robertson, N Sattar, K Shah, J Valabhji, B Young, L S Howard, Mark Toshner, C Berry, P Chowienczyk, A Lawrie, O C Leavy, J Mitchell, J Newman, L Price, J Quint, A Reddy, J Rossdale, N Sattar, C Sudlow, A A R Thompson, J M Wild, M Wilkins, S J Singh, W D-C Man, J M Lord, N J Greening, T Chalder, J T Scott, N Armstrong, E Baldry, M Baldwin, N Basu, M Beadsworth, L Bishop, C E Bolton, A Briggs, M Buch, G Carson, J Cavanagh, H Chinoy, C Dawson, E Daynes, S Defres, R A Evans, L Gardiner, P Greenhaff, S Greenwood, M Harvie, L HOuchen-Wolloff, M Husain, S MacDonald, A McArdle, H J C McAuley, A McMahon, M McNarry, G Mills, C Nolan, K O'Donnell, D Parekh, Pimm, J Sargent, L Sigfrid, M Steiner, D Stensel, A L Tan, I Vogiatzis, J Whitney, D Wilkinson, D Wilson, M Witham, D G Wootton, T Yates, D Thomas, N Brunskill, S Francis, S Greenwood, C Laing, K Bramham, P Chowdhury, A Frankel, L Lightstone, S McAdoo, K McCafferty, M Ostermann, N Selby, C Sharpe, M Willicombe, L Houchen-Wolloff, J Bunker, R Gill, C Hastie, R Nathu, N Rogers, N Smith, A Shaw, L Armstrong, B Hairsine, H Henson, C Kurasz, L Shenton, S Fairbairn, A Dell, N Hawkings, J Haworth, M Hoare, A Lucey, V Lewis, G Mallison, H Nassa, C Pennington, A Price, C Price, A Storrie, G Willis, S Young, P Pfeffer, K Chong-James, C David, W Y James, C Manisty, A Martineau, O Zongo, A Sanderson, L G Heaney, C Armour, V Brown, T Craig, S Drain, B King, N Magee, D McAulay, E Major, L McGarvey, J McGinness, R Stone, A Haggar, A Bolger, F Davies, J Lewis, A Lloyd, R Manley, E McIvor, D Menzies, K Roberts, W Saxon, D Southern, C Subbe, V Whitehead, H El-Taweel, J Dawson, L Robinson, D Saralaya, L Brear, K Regan, K Storton, J Fuld, A Bermperi, I Cruz, K Dempsey, A Elmer, H Jones, S Jose, S Marciniak, M Parkes, C Ribeiro, J Taylor, M Toshner, L Watson, J Weir McCall, J Worsley, R Sabit, L Broad, A Buttress, T Evans, M Haynes, L Jones, L Knibbs, A McQueen, C Oliver, K Paradowski, J Williams, E Harris, C Sampson, C Lynch, E Davies, C Evenden, A Hancock, K Hancock, M Rees, L Roche, N Stroud, T Thomas-Woods, M Babores, J Bradley-Potts, M Holland, N Keenan, S Shashaa, H Wassall, E Beranova, H Weston, T Cosier, L Austin, J Deery, T Hazelton, C Price, H Ramos, R Solly, S Turney, L Pearce, W McCormick, S Pugmire, W Stoker, A Wilson, N Hart, L A Aguilar Jimenez, G Arbane, S Betts, K Bisnauthsing, A Dewar, P Chowdhury, A Chiribiri, A Dewar, G Kaltsakas, H Kerslake, M M Magtoto, P Marino, L M Martinez, C O'Brien, M Ostermann, J Rossdale, T S Solano, E Wynn, N Williams, W Storrar, M Alvarez Corral, A Arias, E Bevan, D Griffin, J Martin, J Owen, S Payne, A Prabhu, A Reed, C Wrey Brown, C Lawson, T Burdett, J Featherstone, A Layton, C Mills, L Stephenson, N Easom, P Atkin, K Brindle, M G Crooks, K Drury, R Flockton, L Holdsworth, A Richards, D L Sykes, S Thackray-Nocera, C Wright, K E Lewis, A Mohamed, G Ross, S Coetzee, K Davies, R Hughes, R Loosley, L O'Brien, Z Omar, H McGuinness, E Perkins, J Phipps, A Taylor, H Tench, R Wolf-Roberts, L S Howard, O Kon, D C Thomas, S Anifowose, L Burden, E Calvelo, B Card, C Carr, E R Chilvers, D Copeland, P Cullinan, P Daly, L Evison, T Fayzan, H Gordon, S Haq, R G Jenkins, C King, K March, M Mariveles, L McLeavey, N Mohamed, S Moriera, U Munawar, J Nunag, U Nwanguma, L Orriss- Dib, D P O'Regan, A Ross, M Roy, E Russell, K Samuel, J Schronce, N Simpson, L Tarusan, C Wood, N Yasmin, R Reddy, A-M Guerdette, M Hewitt, K Warwick, S White, A M Shah, C J Jolley, O Adeyemi, R Adrego, H Assefa-Kebede, J Breeze, M Brown, S Byrne, T Chalder, A Chiribiri, P Dulawan, N Hart, A Hayday, A Hoare, A Knighton, M Malim, C O'Brien, S Patale, I Peralta, N Powell, A Ramos, K Shevket, F Speranza, A Te, P Beirne, A Ashworth, J Clarke, C Coupland, M Dalton, E Wade, C Favager, J Greenwood, J Glossop, L Hall, T Hardy, A Humphries, J Murira, D Peckham, S Plein, J Rangeley, G Saalmink, A L Tan, B Whittam, N Window, J Woods, G Coakley, D G Wootton, L Turtle, L Allerton, A M All, M Beadsworth, A Berridge, J Brown, S Cooper, A Cross, D J Cuthbertson, S Defres, S L Dobson, J Earley, N French, W Greenhalf, H E Hardwick, K Hainey, J Hawkes, V Highett, S Kaprowska, G J Kemp, A L Key, S Koprowska, L Lavelle-Langham, N Lewis-Burke, G Madzamba, F Malein, S Marsh, C Mears, L Melling, M J Noonan, L Poll, J Pratt, E Richardson, A Rowe, M G Semple, V Shaw, K A Tripp, B Vinson, L O Wajero, S A Williams-Howard, J Wyles, S N Diwanji, P Papineni, S Gurram, S Quaid, G F Tiongson, E Watson, B Al-Sheklly, A Horsley, C Avram, J Blaikely, M Buch, N Choudhury, D Faluyi, T Felton, T Gorsuch, N A Hanley, T Hussell, Z Kausar, C A Miller, N Odell, R Osbourne, K Piper Hanley, K Radhakrishnan, S Stockdale, A De Soyza, C Echevarria, A Ayoub, J Brown, G Burns, G Davies, H Fisher, C Francis, A Greenhalgh, P Hogarth, J Hughes, K Jiwa, G Jones, G MacGowan, D Price, A Sayer, J Simpson, H Tedd, S Thomas, S West, M Witham, S Wright, A Young, M J McMahon, P Neill, D Anderson, H Bayes, C Berry, D Grieve, I B McInnes, N Basu, A Brown, A Dougherty, K Fallon, L Gilmour, K Mangion, A Morrow, K Scott, R Sykes, R Touyz, E K Sage, F Barrett, A Donaldson, M Patel, D Bell, A Brown, M Brown, R Hamil, K Leitch, L Macliver, J Quigley, A Smith, B Welsh, G Choudhury, J K Baillie, S Clohisey, A Deans, A B Docherty, J Furniss, E M Harrison, S Kelly, N I Lone, D E Newby, A Sheikh, J D Chalmers, D Connell, A Elliott, C Deas, J George, S Mohammed, J Rowland, A R Solstice, D Sutherland, C J Tee, N Maskell, D Arnold, S Barrett, H Adamali, A Dipper, S Dunn, A Morley, L Morrison, L Stadon, S Waterson, H Welch, B Jayaraman, T Light, C E Bolton, P Almeida, J Bonnington, M Chrystal, E Cox, C Dupont, S Francis, P Greenhaff, A Gupta, L Howard, W Jang, S Linford, L Matthews, R Needham, A Nikolaidis, S Prosper, K Shaw, A K Thomas, L P Ho, N M Rahman, M Ainsworth, A Alamoudi, M Beggs, A Bates, A Bloss, A Burns, P Carter, M Cassar, K M Channon, J Chen, F Conneh, T Dong, R I Evans, E Fraser, X Fu, J R Geddes, F Gleeson, P Harrison, M Havinden-Williams, P Jezzard, N Kanellakis, I Koychev, P Kurupati, X Li, E Lukaschuk, K McGlynn, H McShane, C Megson, K Motohashi, S Neubauer, D Nicoll, G Ogg, E Pacpaco, M Pavlides, Y Peng, N Petousi, J Propescu, N Rahman, B Raman, M J Rowland, K Saunders, M Sharpe, N Talbot, E Tunnicliffe, W D- C Man, B Patel, R E Barker, D Cristiano, N Dormand, M Gummadi, S Kon, K Liyanage, C M Nolan, S Patel, O Polgar, P Shah, S J Singh, J A Walsh, J R Hurst, H Jarvis, S Mandal, S Ahmad, S Brill, L Lim, D Matila, O Olaosebikan, C Singh, M Toshner, H Baxendale, L Garner, C Johnson, J Mackie, A Michael, J Pack, K Paques, H Parfrey, J Parmar, N Diar Bakerly, P Dark, D Evans, E Hardy, A Harvey, D Holgate, S Knight, N Mairs, N Majeed, L McMorrow, J Oxton, J Pendlebury, C Summersgill, R Ugwuoke, S Whittaker, W Matimba-Mupaya, S Strong-Sheldrake, S L Rowland-Jones, A A R Thompson, J Bagshaw, M Begum, K Birchall, R Butcher, H Carborn, F Chan, K Chapman, Y Cheng, L Chetham, C Clark, Z Coburn, J Cole, M Dixon, A Fairman, J Finnigan, L Finnigan, H Foot, D Foote, A Ford, R Gregory, K Harrington, L Haslam, L Hesselden, J Hockridge, A Holbourn, B Holroyd-Hind, L Holt, A Howell, E Hurditch, F Ilyas, C Jarman, A Lawrie, E Lee, J- H Lee, R Lenagh, A Lye, I Macharia, M Marshall, A Mbuyisa, J McNeill, S Megson, J Meiring, L Milner, S Misra, H Newell, T Newman, C Norman, L Nwafor, D Pattenadk, M Plowright, J Porter, P Ravencroft, C Roddis, J Rodger, P Saunders, J Sidebottom, J Smith, L Smith, N Steele, G Stephens, R Stimpson, B Thamu, N Tinker, K Turner, H Turton, P Wade, S Walker, J Watson, J M Wild, I Wilson, A Zawia, R Aul, M Ali, A Dunleavy, D Forton, N Msimanga, M Mencias, T Samakomva, S Siddique, J Teixeira, V Tavoukjian, J Hutchinson, L Allsop, K Bennett, P Buckley, M Flynn, M Gill, C Goodwin, M Greatorex, H Gregory, C Heeley, L Holloway, M Holmes, J Kirk, W Lovegrove, T A Sewell, S Shelton, D Sissons, K Slack, S Smith, D Sowter, S Turner, V Whitworth, I Wynter, L Warburton, S Painter, J Tomlinson, C Vickers, T Wainwright, D Redwood, J Tilley, S Palmer, G A Davies, L Connor, A Cook, T Rees, F Thaivalappil, C Thomas, A Butt, M Coulding, H Jones, S Kilroy, J McCormick, J McIntosh, H Savill, V Turner, J Vere, E Fraile, J Ugoji, S S Kon, H Lota, G Landers, M Nasseri, S Portukhay, A Hormis, A Daniels, J Ingham, L Zeidan, M Chablani, L Osborne, M Marks, J S Brown, N Ahwireng, B Bang, D Basire, R C Chambers, A Checkley, R Evans, M Heightman, T Hillman, J Hurst, J Jacob, S Janes, R Jastrub, M Lipman, S Logan, D Lomas, M Merida Morillas, A Pakzad, H Plant, J C Porter, K Roy, E Wall, B Williams, M Xu, D Parekh, N Ahmad Haider, C Atkin, R Baggott, M Bates, A Botkai, A Casey, B Cooper, J Dasgin, K Draxlbauer, N Gautam, J Hazeldine, T Hiwot, S Holden, K Isaacs, T Jackson, S Johnson, V Kamwa, D Lewis, J M Lord, S Madathil, C McGhee, K Mcgee, A Neal, A Newton Cox, J Nyaboko, D Parekh, Z Peterkin, H Qureshi, B Rangelov, L Ratcliffe, E Sapey, J Short, T Soulsby, R Steeds, J Stockley, Z Suleiman, T Thompson, M Ventura, S Walder, C Welch, D Wilson, S Yasmin, K P Yip, P Beckett, C Dickens, U Nanda, C E Brightling, R A Evans, M Aljaroof, N Armstrong, H Arnold, H Aung, M Bakali, M Bakau, M Baldwin, M Bingham, M Bourne, C Bourne, N Brunskill, P Cairns, L Carr, A Charalambou, C Christie, M J Davies, S Diver, S Edwards, C Edwardson, O Elneima, H Evans, J Finch, S Glover, N Goodman, B Gootpu, N J Greening, K Hadley, P Haldar, B Hargadon, V C Harris, L Houchen-Wolloff, W Ibrahim, L Ingram, K Khunti, A Lea, D Lee, G P McCann, H J C McAuley, P McCourt, T Mcnally, G Mills, A Moss, W Monteiro, M Pareek, S Parker, A Rowland, A Prickett, I N Qureshi, R J Russell, N Samani, M Sereno, M Sharma, A Shikotra, S Siddiqui, A Singapuri, S J Singh, J Skeemer, M Soares, E Stringer, T Thornton, M Tobin, E Turner, L V Wain, T J C Ward, F Woodhead, J Wormleighton, T Yates, A Yousuf, M G Jones, C Childs, R Djukanovic, S Fletcher, M Harvey, E Marouzet, B Marshall, R Samuel, T Sass, T Wallis, H Wheeler, R Dharmagunawardena, E Bright, P Crisp, M Stern, A Wight, L Bailey, A Reddington, A Ashish, J Cooper, E Robinson, A Broadley, K Howard, L Barman, C Brookes, K Elliott, L Griffiths, Z Guy, D Ionita, H Redfearn, C Sarginson, A Turnbull, Y Ellis, M Marks, A Briggs, K Holmes, K Poinasamy, S Walker, M Halling-Brown, G Breen, M Hotopf, K Lewis, N Williams

**Affiliations:** The Institute for Lung Health, NIHR Leicester Biomedical Research Centre-Respiratory, University of Leicester, Leicester, UK; The Institute for Lung Health, NIHR Leicester Biomedical Research Centre-Respiratory, University of Leicester, Leicester, UK; The Institute for Lung Health, NIHR Leicester Biomedical Research Centre-Respiratory, University of Leicester, Leicester, UK; Department of Population Health Sciences, University of Leicester, Leicester, UK; University of Dundee, Ninewells Hospital and Medical School, Dundee, UK; Division of Infection, Immunity & Respiratory Medicine, Faculty of Biology, Medicine and Health, University of Manchester, Manchester, UK; Manchester University NHS Foundation Trust, Manchester, UK; MRC Human Immunology Unit, University of Oxford, Oxford, UK; Oxford University Hospitals NHS Foundation Trust, Oxford, UK; Department of Clinical Research, London School of Hygiene & Tropical Medicine, London, UK; Hospital for Tropical Diseases, University College London Hospital, London, UK; Asthma and Lung UK, London, UK; Oxford University Hospitals NHS Foundation Trust, Oxford, UK; Division of Cardiovascular Medicine, Radcliffe Department of Medicine, University of Oxford, Oxford, UK; The Institute for Lung Health, NIHR Leicester Biomedical Research Centre-Respiratory, University of Leicester, Leicester, UK; The Institute for Lung Health, NIHR Leicester Biomedical Research Centre-Respiratory, University of Leicester, Leicester, UK; The Institute for Lung Health, NIHR Leicester Biomedical Research Centre-Respiratory, University of Leicester, Leicester, UK; The Institute for Lung Health, NIHR Leicester Biomedical Research Centre-Respiratory, University of Leicester, Leicester, UK; Centre for Exercise and Rehabilitation Science, NIHR Leicester Biomedical Research Centre-Respiratory, University of Leicester, Leicester, UK; Department of Respiratory Sciences, University of Leicester, Leicester, UK; The Institute for Lung Health, NIHR Leicester Biomedical Research Centre-Respiratory, University of Leicester, Leicester, UK; The Institute for Lung Health, NIHR Leicester Biomedical Research Centre-Respiratory, University of Leicester, Leicester, UK; The Institute for Lung Health, NIHR Leicester Biomedical Research Centre-Respiratory, University of Leicester, Leicester, UK; National Heart and Lung Institute, Imperial College London, London, UK; London School of Hygiene & Tropical Medicine, London, UK; Centre for Medical Informatics, The Usher Institute, University of Edinburgh, Edinburgh, UK; Centre for Medical Informatics, The Usher Institute, University of Edinburgh, Edinburgh, UK; The Roslin Institute, University of Edinburgh, Edinburgh, UK; Centre for Medical Informatics, The Usher Institute, University of Edinburgh, Edinburgh, UK; Centre for Medical Informatics, The Usher Institute, University of Edinburgh, Edinburgh, UK; Royal Infirmary of Edinburgh, NHS Lothian, Edinburgh, UK; Centre for Medical Informatics, The Usher Institute, University of Edinburgh, Edinburgh, UK; Wellcome-Wolfson Institute for Experimental Medicine, Queens University Belfast, Belfast, UK; Belfast Health & Social Care Trust, Belfast, UK; Hywel Dda University Health Board, Wales, UK; University of Swansea, Swansea, Wales, UK; St George's University Hospitals NHS Foundation Trust, London, UK; Leeds Teaching Hospitals NHS Trust, Leeds, UK; Nottingham University Hospitals NHS Trust, Nottingham, UK; NIHR Nottingham Biomedical Research Centre, University of Nottingham, Nottingham, UK; UCL Respiratory, Department of Medicine, University College London, London, UK; Royal Infirmary of Edinburgh, NHS Lothian, Edinburgh, UK; Centre for Inflammation Research, University of Edinburgh, Edinburgh, UK; Manchester Metropolitan University, Manchester, UK; Salford Royal NHS Foundation Trust, Manchester, UK; Infection Research Group, Hull University Teaching Hospitals, Hull, UK; University of Hull, Hull, UK; Newcastle Upon Tyne Hospitals NHS Foundation Trust, Newcastle Upon Tyne, UK; Translational and Clinical Research Institute, Newcastle University, Newcastle Upon Tyne, UK; Department of Respiratory Medicine, Cambridge University Hospitals NHS Foundation Trust, Cambridge, UK; NIHR Cambridge Clinical Research Facility, Cambridge, UK; Lane Fox Respiratory Unit, Guy’s and St Thomas’ NHS Foundation Trust, London, UK; UCL Respiratory, Department of Medicine, University College London, London, UK; Royal Free London NHS Foundation Trust, London, UK; Clinical and Experimental Sciences, Faculty of Medicine, University of Southampton, Southampton, UK; NIHR Southampton Biomedical Research Centre, Southampton, UK; Institute of Inflammation and Ageing, University of Birmingham, Birmingham, UK; University Hospital Birmingham NHS Foundation Trust, Birmingham, UK; Department of Respiratory Medicine, Barts Health NHS Trust, London, UK; Barts and The London School of Medicine and Dentistry, Queen Mary University of London, London, UK; Oxford University Hospitals NHS Foundation Trust, Oxford, UK; NIHR Oxford Biomedical Research Centre, Oxford, UK; University of Sheffield, Sheffield, UK; Sheffield Teaching Hospitals NHS Foundation Trust, Sheffield, UK; University of Sheffield, Sheffield, UK; Sheffield Teaching Hospitals NHS Foundation Trust, Sheffield, UK; Centre for Human & Applied Physiological Sciences, School of Basic & Medical Biosciences, Faculty of Life Sciences & Medicine, King’s College London, London, UK; King’s College Hospital NHS Foundation Trust, London, UK; King’s College Hospital NHS Foundation Trust, London, UK; King’s College London British Heart Foundation Centre, London, UK; NIHR Health Protection Research Unit in Emerging and Zoonotic Infections, University of Liverpool, Liverpool, UK; Liverpool University Hospitals NHS Foundation Trust, Liverpool, UK; Department of Psychological Medicine, Institute of Psychiatry, Psychology and Neuroscience, King's College London, London, UK; South London and Maudsley NHS Foundation Trust, London, UK; Diabetes Research Centre, University of Leicester, Leicester, UK; NIHR Leicester Biomedical Research Centre, University of Leicester, Leicester, UK; Newcastle Upon Tyne Hospitals NHS Foundation Trust, Newcastle Upon Tyne, UK; Population Health Sciences Institute, Newcastle University, Newcastle Upon Tyne, UK; Oxford University Hospitals NHS Foundation Trust, Oxford, UK; NIHR Oxford Health Biomedical Research Centre, University of Oxford, Oxford, UK; Liverpool University Hospitals NHS Foundation Trust, Liverpool, UK; The CRUK Liverpool Experimental Cancer Medicine Centre, Liverpool, UK; Department of Oncology and Metabolism, University of Sheffield, Sheffield, UK; National Heart and Lung Institute, Imperial College London, London, UK; Imperial College Healthcare NHS Trust, London, UK; Centre for Medical Image Computing, University College London, London, UK; Lungs for Living Research Centre, University College London, London, UK; National Heart and Lung Institute, Imperial College London, London, UK; MRC-Versus Arthritis Centre for Musculoskeletal Ageing Research, Institute of Inflammation and Ageing, University of Birmingham, Birmingham, UK; NIHR Birmingham Biomedical Research Centre, Birmingham, UK; Royal Brompton & Harefield Hospitals, Guy’s and St. Thomas’ NHS Foundation Trust, London, UK; Faculty of Life Sciences & Medicine, King’s College London, London, UK; NIHR Leicester Biomedical Research Centre, University of Leicester, Leicester, UK; Department of Cardiovascular Sciences, University of Leicester, Leicester, UK; Division of Cardiovascular Medicine, Radcliffe Department of Medicine, University of Oxford, Oxford, UK; NIHR Oxford Biomedical Research Centre, Oxford, UK; National Heart and Lung Institute, Imperial College London, London, UK; UCL Respiratory, Department of Medicine, University College London, London, UK; Kadoorie Centre for Critical Care Research, Nuffield Department of Clinical Neurosciences, University of Oxford, Oxford, UK; MRC-University of Glasgow Centre for Virus Research, Glasgow, UK; NIHR Health Protection Research Unit in Emerging and Zoonotic Infections, University of Liverpool, Liverpool, UK; Respiratory Medicine, Alder Hey Children’s Hospital, Liverpool, UK; The Institute for Lung Health, NIHR Leicester Biomedical Research Centre-Respiratory, University of Leicester, Leicester, UK; Centre for Exercise and Rehabilitation Science, NIHR Leicester Biomedical Research Centre-Respiratory, University of Leicester, Leicester, UK; Department of Immunology and Inflammation, Imperial College London, London, UK; NIHR Cambridge Clinical Research Facility, Cambridge, UK; NIHR Cambridge Biomedical Research Centre, Cambridge, UK; Founding Member of Long Covid Support, Windsor, UK; Centre for Medical Informatics, The Usher Institute, University of Edinburgh, Edinburgh, UK; The Institute for Lung Health, NIHR Leicester Biomedical Research Centre-Respiratory, University of Leicester, Leicester, UK; The Institute for Lung Health, NIHR Leicester Biomedical Research Centre-Respiratory, University of Leicester, Leicester, UK; Department of Population Health Sciences, University of Leicester, Leicester, UK; The Institute for Lung Health, NIHR Leicester Biomedical Research Centre-Respiratory, University of Leicester, Leicester, UK

**Keywords:** COVID-19, comorbidities, symptoms

Key Features

- The Post-Hospitalisation COVID-19 (PHOSP-COVID) study is a national UK multicentre cohort study of patients who were hospitalized for COVID-19 and subsequently discharged.
- PHOSP-COVID was established to investigate the medium- and long-term sequelae of severe COVID-19 requiring hospitalization, understand the underlying mechanisms of these sequelae, evaluate the medium- and long-term effects of COVID-19 treatments and to serve as a platform to enable future studies, including clinical trials.
- Data collected covered a wide range of physical measures, biological samples and patient-reported outcome measures (PROMs).
- Participants could join the cohort either in Tier 1 only with remote data collection using hospital records, a PROMs app and postal saliva sample for DNA; or in Tier 2 in which they were invited to attend two specific research visits for further data collection and biological research sampling. These research visits occurred at 5 (range 2–7) months and 12 (range 10–14) months post-discharge. Participants could also participate in specific nested studies (Tier 3) at selected sites.
- All participants were asked to consent to further follow-up for 25 years via linkage to their electronic healthcare records and to be re-contacted for further research.
- In total, 7935 participants were recruited from 83 UK sites: 5238 to Tier 1 and 2697 to Tier 2, between August 2020 and March 2022.
- Cohort data are held in a Trusted Research Environment and samples stored in a central biobank. Data and samples can be accessed upon request and subject to approvals from https://www.phosp.org/data-sample-request/.

## Why was the cohort set up?

To date, there have been >750 million reported cases of COVID-19 globally since the pandemic began in early 2020.^1^ In the UK, there have been >1 million patients hospitalized and 180 000 deaths due to COVID-19.^2^ Previous viral epidemics and conditions causing acute respiratory distress syndrome caused long-lasting health impacts on the affected survivors.^3^^,^^4^ At the time of conception of the Post-Hospitalisation COVID-19 (PHOSP-COVID) cohort in March 2020, the longer-term pulmonary and multisystem effects of COVID-19 and impact on health status were unknown.^5^ We identified a need to establish a cohort of hospitalized COVID-19 survivors to collect detailed information about the medium- and long-term effects of COVID-19 on physical and mental health, lifestyle and occupation status.

Although the majority of individuals with COVID-19 were not hospitalized, we expected that the consequences of COVID-19 might be most pronounced after severe illness. Furthermore, the pressures on health systems during the pandemic needed to be taken into consideration when establishing a new clinical cohort. Therefore, we designed the PHOSP-COVID study to align with clinical follow-up reviews of hospitalized patients, where possible.

PHOSP-COVID was designed to take a patient-centred, holistic approach to understanding the medium- and long-term effects of COVID-19, recognizing the need to consider physical and mental health, social support and lifestyle. There were three main aims of PHOSP-COVID:

- To determine the medium- and long-term health (and health economic) sequelae of COVID-19 in post-hospitalization survivors; to define demographic, clinical and molecular biomarkers of susceptibility, including to severity of the acute illness and development, progression and resolution of sequelae.
- To understand the impact of inpatient and post-discharge, pharmacological and non-pharmacological interventions on long-term sequelae of COVID-19.
- To build the foundation for in-depth studies of emergent conditions and worsening of pre-morbid disease to inform precision medicine in at-risk groups by directing new clinical trials and care for current and future patients with long COVID.

## Who is in the cohort?

Individuals who were discharged from hospital between 1 February 2020 and 31 March 2021 were invited to participate in the PHOSP-COVID study if they were: aged ≥18 years, admitted to a participating UK hospital with confirmed or clinically suspected COVID-19 and able to provide informed consent either personally or via a consultee or an appropriate representative. Exclusion criteria included: admission due to a diagnosis of a different pathogen with no indication or likelihood of co-infection with COVID-19, attendance at emergency department only, declined to provide informed consent or life-limiting illness with life expectancy of <6 months such as disseminated malignancy. During the recruitment period (August 2020 to March 2022), eligible patients were invited to participate in the study by research teams based at the participating sites ≤1 year after discharge. A total of 83 sites from England, Northern Ireland, Scotland and Wales participated following the study advertisement in social media and research networks. Different methods were used to obtain consent including: face-to-face, telephone, postal and eConsent.

**Figure 1.**
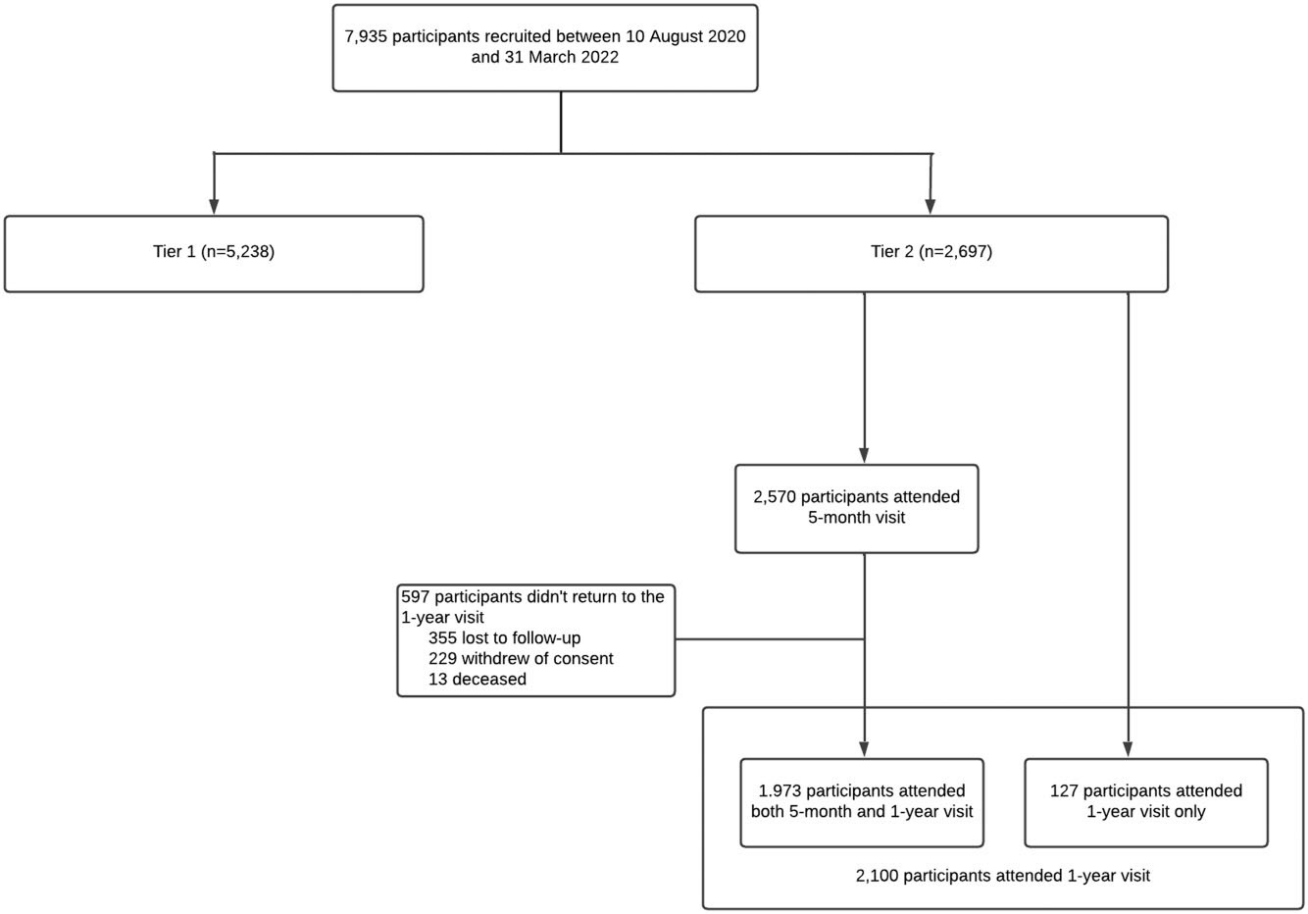
Consort diagram of the Post-Hospitalisation COVID-19 (PHOSP-COVID) study. ^a^The wide range window for the first research visit (2–7 months) was deliberately chosen to accommodate the variation in planned clinical follow-up appointments across the different participating sites and to allow the research visit to coincide with the planned clinical follow-up appointments

Participants could join as Tier 1 participants only with remote data collection or could join as Tier 2 participants in which they were invited to attend two research visits for further data collection and biological research sampling (Figure 1).

Participants in either Tier 1 or Tier 2 could additionally join Tier 3 sub-studies in which they were either recalled for additional research procedures or undertook additional research procedures during their Tier 2 research visits. For example, a subset of 141 participants had an extended blood draw to enable additional sampling and advanced cellular studies^6^ and another subset of 531 participants completed up to three whole-body magnetic resonance imaging (MRI) scans to examine the effect of COVID-19 on multiple body organs (Capturing MultiORgan Effects of COVID-19, C-MORE sub-study).^7^^,^^8^

A total of 7935 participants were recruited into the PHOSP-COVID cohort—5238 participants to Tier 1 and 2697 to Tier 2—between 10 August 2020 and 31 March 2022. The participants’ demographics, comorbidities and admission characteristics are detailed in Table 1 and Supplementary Table S1 (available as Supplementary data at *IJE* online). Over 1000 participants to date have also been included in Tier 3 studies.

**Table 1.** Participant demographics, comorbidities and admission characteristics of the Post-Hospitalisation COVID-19 (PHOSP-COVID) cohort

**Characteristic** ^a^	Complete PHOSP-COVID cohort (*N*=7935)		Tier 1 (*n*=5238)		Tier 2 (*n*=2697)	
	*n*	Value	*n*	Value	*n*	Value
Age at admission (years)^b^	7926	59.3 (13.4)	5230	59.9 (13.8)	2696	58.0 (12.6)
Missing data		9 (0.1%)		8 (0.2%)		1 (<0.1%)
Sex	7926		5230		2696	
Female		3206 (40.4%)		2168 (41.5%)		1038 (38.5%)
Male		4720 (59.6%)		3062 (58.5%)		1658 (61.5%)
Missing data		9 (0.1%)		8 (0.2%)		1 (<0.1%)
Ethnicity	7697		5019		2678	
White		6298 (81.8%)		4291 (85.5%)		2007 (74.9%)
South Asian		629 (8.2%)		324 (6.5%)		305 (11.4%)
Black		375 (4.9%)		182 (3.6%)		193 (7.2%)
Mixed		120 (1.5%)		65 (1.3%)		55 (2.1%)
Other		275 (3.6%)		157 (3.1%)		118 (4.4%)
Missing data		238 (3.0%)		219 (4.2%)		19 (0.7%)
Index of Multiple Deprivation score	7869		5192		2677	
1 (most deprived)		1810 (23.0%)		1192 (23.0%)		618 (23.1%)
2		1717 (21.8%)		1095 (21.1%)		622 (23.2%)
3		1407 (17.9%)		944 (18.2%)		463 (17.3%)
4		1496 (19.0%)		1024 (19.7%)		472 (17.6%)
5 (least deprived)		1439 (18.3%)		937 (18.0%)		502 (18.8%)
Missing data		66 (0.8%)		46 (0.9%)		20 (0.7%)
Body mass index	2693		417		2276	
Median^c^		31.2 [27.6–36.1]		31.8 [27.2–36.8]		31.2 [27.7–36.0]
<30 kg/m^2^		1121 (41.6%)		169 (40.5%)		952 (41.8%)
≥30 kg/m^2^		1572 (58.4%)		248 (59.5%)		1324 (58.2%)
Missing data		5242 (66.1%)		4821 (92.0%)		421 (15.6%)
Healthcare worker	7175	879 (12.3%)	4620	503 (10.9%)	2555	376 (14.7%)
Missing data		760 (9.6%)		618 (11.8%)		142 (5.2%)
Admission duration (days)^b^	7935	13.5 (17.5)	5238	13.4 (17.2)	2697	14.1 (17.9)
WHO clinical progression scale^d^	7927		5230		2697	
WHO Class 3–4		1361 (17.2%)		914 (17.5%)		447 (16.6%)
WHO Class 5		3530 (44.5%)		2395 (45.8%)		1135 (42.0%)
WHO Class 6		1938 (24.4%)		1305 (24.9%)		633 (23.5%)
WHO Class 7–9		1098 (13.9%)		616 (11.8%)		482 (17.9%)
Missing data		8 (0.1%)		8 (0.2%)		0
Comorbidities	7935		5238		2697	
Median number of comorbidities^c^		2 [1–3]		2 [1–3]		2 [1–3]
0		1792 (22.6%)		1125 (21.5%)		667 (24.7%)
1		1721 (21.7%)		1150 (21.9%)		571 (21.2%)
≥2		4422 (55.7%)		2963 (56.6%)		1459 (54.1%)
Cardiovascular	7935	3763 (47.4%)	5238	2524 (48.2%)	2697	1239 (45.9%)
Respiratory	7935	2282 (28.8%)	5238	1558 (29.7%)	2697	724 (26.8%)
Neuro-psychiatric	7935	1689 (21.3%)	5238	1127 (21.5%)	2697	562 (20.8%)
Renal and endocrine	7935	959 (12.1%)	5238	672 (12.8%)	2697	287 (10.6%)
Type 2 diabetes	7913	1683 (21.3%)	5222	1146 (21.9%)	2691	537 (19.9%)
Missing data		22 (0.3%)		16 (0.3%)		6 (0.2%)
Positive SARS-CoV-2 PCR	7309	6840 (93.6%)	4842	4557 (94.1%)	2467	2283 (92.5%)
Missing data		626 (7.9%)		396 (7.6%)		230 (8.5%)
Systemic steroids	7529	4602 (61.1%)	4968	3154 (63.5%)	2561	1448 (65.5%)
Missing data		406 (5.1%)		270 (5.2%)		136 (5.1%)
Antibiotic therapy	7719	6161 (79.8%)	5087	4086 (80.3%)	2632	2075 (78.8%)
Missing data		216 (2.7%)		151 (2.9%)		65 (2.4%)
Anticoagulants	7461	3616 (48.5%)	4896	2443 (49.9%)	2565	1173 (45.7%)
Missing data		474 (5.9%)		342 (6.5%)		132 (4.9%)

a Data are *n* (%) unless indicated. Percentages are calculated by category after exclusion of missing data for that variable.

b Mean (SD).

c Median [IQR].

d WHO classes are: 3–4 = no continuous supplemental oxygen needed; 5 = continuous supplemental oxygen only; 6 = continuous or bi-level positive airway pressure ventilation or high-flow nasal oxygen; and 7–9 = invasive mechanical ventilation or other organ support.

See Supplementary Table SM1 (available as Supplementary data at *IJE* online) for further descriptions of variables.

IQR, interquartile range; SARS-CoV-2 PCR, severe acute respiratory syndrome coronavirus 2 polymerase chain reaction; WHO, World Health Organization.

Overall, the cohort has a mean age of 59.3 years, 40% of participants are female, 82% report White ethnicity and 23% are from the lowest quintile of the Index of Multiple Deprivation. The cohort was comorbid, with >55% of participants having two or more pre-existing comorbidities at the time of hospital admission. More than 93% had a positive SARS-CoV-2 RT-PCR test result on admission and 38% required non-invasive or invasive ventilation (Class 6 or above on the World Health Organization clinical progression scale)^9^ during their original hospital admission.

Given the pressures of the ongoing pandemic during recruitment, non-response to invitations to join the study was not recorded.

## How often have they been followed up?

Data collection for Tier 1 participants was restricted to available clinical data from routine hospital follow-up plus the collection of patient-reported outcome measures (PROMs) via an app every 3 months for ≤1 year post discharge. Tier 2 participants were invited to two research visits: the first between 2 and 7 months, and the second between 10 and 14 months post hospital discharge. Of the 2570 Tier 2 participants who attended the first research visit (labelled as the 5-month visit due to the median length of time between discharge and the visit), 1973 participants also attended a second research visit (labelled the 1-year visit). A further 127 Tier 2 participants attended the 1-year visit only (Figure 1). The characteristics of the 597 participants who did not return for a 1-year visit are listed in Supplementary Table S2 (available as Supplementary data at *IJE* online).

All participants provided consent for further data collection via linkage to retrospective and prospective healthcare and social-care records including primary care, hospital episode statistics and specialist tertiary clinical databases for ≤25 years. Participants were also invited to provide consent to be re-contacted for further research, including Tier 3 sub-studies, such as mechanistic studies and clinical trials.^10^

## What has been measured?

A summary of the data collected for PHOSP-COVID participants is provided in Table 2. For all participants, information about their demographics, acute illness and hospital admission were obtained retrospectively from hospital notes by the research team once a consent form was signed. This included: comorbidities, presenting symptoms, length of stay, severity of acute illness, treatment received, complications and common clinical test results. Hospital records were also reviewed to collect clinical data obtained from any planned follow-up appointments organized by the local hospital team after discharge. These included: physiological tests and imaging, routine blood test results and clinical questionnaires (Supplementary Table SM1, available as Supplementary data at *IJE* online). Further data were collected on post-discharge care accessed including mental health interventions, rehabilitation programmes and details from any emergency hospital admission for ≤1 year post discharge. All the captured data measures were recorded on paper forms then transferred to a study-specific online database and subsequently to a national Data Safe Haven.

**Table 2.** The Post-Hospitalisation COVID-19 (PHOSP-COVID) outcome measures

Module	Details	Tier 1	Tier 2	Tier 3
Time point: Hospital discharge				
Baseline demographics	Age, sex at birth, ethnicity, education, household income	✓	✓	
	Occupation (including changes after hospitalization)	✓	✓	
	Smoking and alcohol consumption	✓	✓	
	Index of Multiple Deprivation score	✓	✓	
	Clinical comorbidities	✓	✓	
Hospitalization details	Length of stay	✓	✓	
	Presenting symptoms/signs and duration	✓	✓	
	Vital signs at admission	✓	✓	
	Level of respiratory and other organs support	✓	✓	
	Received treatment/intervention	✓	✓	
	Additional diagnoses (e.g. pulmonary embolism, myocarditis)	✓	✓	
	Medications pre-admission and on discharge	✓	✓	
	Enrolment into acute COVID-19 studies	✓	✓	
	Clinical blood results (e.g. FBC, BNP/NT-proBNP, CRP)	✓	✓	
	SARS-CoV-2 Swab PCR status	✓	✓	
**Time points: Research visits at 5 months and 1 year after discharge**				
Clinical assessment at clinical follow-up/research visits	ECG findings	^a^	✓	
	Clinical investigation results: chest X-ray, echocardiogram, FeNO, CPET, 6MWT, etc.	^a^	✓	
	Outcome of clinical review	^a^	✓	
Clinical investigations	Blood: FBC, U&Es, LFTs, eGFR, CRP, bone, vitamin D, troponin, BNP/NT-proBNP, D-dimer, INR, fibrinogen, ferritin, HbA1C, lipid profile	^a^	✓	
	Fasting blood samples: glucose, insulin, fasting lipid profile		✓	
	Urine: urinalysis, albumin: creatinine ratio and protein: creatinine ratio		✓	
Biological samples for research	Blood (serum, plasma, DNA, RNA)		✓	
	Oral rinse		✓	
	Sputum (spontaneous)		✓	
	Urine		✓	
	Blood PBMCs			✓
	Muscle biopsies			✓
	Saliva (DNA)	✓		
Health-related quality of life and disability	EuroQol EQ-5D-5L	^b^	✓	
	Washington Short Set of Functioning (WG-SS-Sco)	^b^	✓	
Patient-reported outcome measures (PROMs)	PHOSP-COVID study-specific tool—Patient Symptom Questionnaire (PSQ)	^b^	✓	
	MRC dyspnoea scale	^b^	✓	
	Dyspnoea12 Questionnaire	^b^	✓	
	Generalized Anxiety Disorder Questionnaire (GAD-7)	^b^	✓	
	Patient Health Questionnaire (PHQ-9)	^b^	✓	
	Functional Assessment of Chronic Illness Therapy—Fatigue Scale (FACIT-Fatigue)	^b^	✓	
	Brief Pain Inventory Questionnaire (BPI)	^b^	✓	
	Nottingham Activities of Daily Living (NEADL) Questionnaire	^b^	✓	
	Post-Traumatic Stress Disorder Checklist for DSM5 Questionnaire (PCL-5)	^b^	✓	
	Sleep questionnaires:			
	Pittsburgh Sleep Quality Index (PSQI)			✓
	Morningness-Eveningness Questionnaire (MEQ)			✓
	Leicester Cough Questionnaire (LCQ)			✓
Cognitive assessment	Montreal Cognitive Assessment (MoCA)	^b^	✓	
	Cognitron online test			✓
Physical activity and performance	General Practice Physical Activity Questionnaire (GPPAQ)		✓	
	Daily physical activity by wearable monitor (GENEactive©)		✓	
	Incremental Shuttle Walk Test (ISWT)		✓	
	Short Physical Performance Battery (SPPB)		✓	
	Handgrip strength		✓	
	Quadriceps muscle strength			✓
Frailty assessment	Rockwood Clinical Frailty Scale (CFS)		✓	
	Fried’s frailty definition		✓	
Body composition	Body mass index	✓	✓	
	SARC-F Questionnaire		✓	
	Waist circumference measurement		✓	
	Bioelectrical impedance analysis (BIA)		✓	
	Dual energy X-ray analysis (DXA)			✓
Pulmonary function tests	Spirometry (FEV1, FVC, FEV1/FVC)		✓	
	Transfer factor (TLCO, KCO)		✓	
	Max inspiratory pressure (MIP)			✓
	Max expiratory pressure (MEP)			✓
Radiological images acquisition	Chest radiograph	^a^	^a^	
	CT thorax	^a^	^a^	
	Multi-organs MRI scan			✓

a The results of these outcomes measures were only available for collection if performed for clinical indications by the local medical team.

b A subset of Tier 1 participants remotely completed health-related questionnaires using an electronic app.

6MWT, 6-min walk test; BNP, brain natriuretic peptide; CPET, cardiopulmonary exercise testing; CRP, C-reactive protein; CT, computed tomography; DNA, deoxyribonucleic acid; ECG, electrocardiogram; eGFR, estimated glomerular filtration rate; FBC, full blood count; FeNO, fractional exhaled nitric oxide; FEV1, forced expiratory volume measured in 1 s; FVC, forced vital capacity; HbA1C, glycated haemoglobin; INR, international normalized ratio; KCO, carbon monoxide transfer coefficient; LFTs, liver function tests; MRC, Medical Research Council; MRI, magnetic resonance imaging; NT-BNP, N-terminal BNP; PBMCs, peripheral blood mononuclear cells; PCR, polymerase chain reaction; RNA, ribonucleic acid; SARS-CoV-2, severe acute respiratory syndrome coronavirus 2; TLCO, transfer capacity of the lung for carbon monoxide; U&Es, urea, creatinine and electrolytes.

For participants in Tier 1, clinical data were obtained from medical records and no specific research visit was undertaken. However, a subset of Tier 1 participants used an online app to remotely complete PROM questionnaires and a bespoke study-specific Patient Symptom Questionnaire (PSQ).^11^ The PSQ was used to collect information about ongoing symptoms, changes in occupation and perceived recovery where the participant was asked to answer ‘yes’, ‘no’ or ‘not sure’ to the question: ‘Do you feel fully recovered from COVID-19?’ A total of 371 participants provided 519 entries using the online PROMs app (142 Tier 1 and 229 Tier 2) between April 2021 and April 2022. Another subset of Tier 1 participants provided a saliva sample for DNA analysis via a collection kit posted to their home (Supplementary Table S3, available as Supplementary data at *IJE* online).

At Tier 2 research visits, clinical questionnaires, procedures and sampling were undertaken including completion of the PSQ. Physical performance was assessed using questionnaires and physical tests including: handgrip and quadriceps strength, Short Physical Performance Battery and Incremental Shuttle Walk Test. All Tier 2 participants were additionally invited to undertake daily physical activity monitoring using a wearable GENEactive© accelerometer for 14 days. Lung function was assessed using spirometry and measurement of gas transfer when feasible given the COVID-19 restrictions on aerosol-generating procedures (Table 3).

**Table 3.** Patient-reported outcome measures, and physiological and biochemical tests among Tier 2 participants stratified by the research visits

	Available data (*n*)	5-month visit (*n*=2570)	Available data (*n*)	1-year visit (*n*=2100)
Time from discharge (days)^a^	2570	158.9 (47.4)	2100	380.9 (35.0)
Recovered from COVID-19?	2202		1787	
Yes		567 (25.7%)		541 (30.3%)
No		1215 (55.2%)		863 (48.3%)
Not sure		420 (19.1%)		383 (21.4%)
Missing data		368 (14.3%)		313 (14.9%)
5-month recovery cluster assignment	2405		1881	
Mild		723 (30.1%)		567 (30.1%)
Moderate/cognitive		543 (22.6%)		426 (22.7%)
Severe		636 (26.4%)		502 (26.7%)
Very severe		503 (20.9%)		386 (20.5%)
Missing data		165 (6.4%)		219 (10.4%)
**PROMs**				
Self-report symptom count^b^	2267	8 [3–13]	1814	9 [4–16]
Missing data		303 (11.8%)		286 (13.6%)
GAD-7 total score^a^	2408	5.35 (5.72)	1950	5.06 (5.65)
Anxiety (GAD-7 > 8)	2408	614 (25.5%)	1950	461 (23.6%)
Missing data		162 (6.3%)		150 (7.1%)
PHQ-9 total score^a^	2406	7.04 (6.57)	1947	6.43 (6.39)
Depression (PHQ-9 ≥ 10)	2406	734 (30.5%)	1947	509 (26.1%)
Missing data		164 (6.4%)		153 (7.3%)
PCL-5 total score^a^	2403	15.84 (17.24)	1937	14.28 (16.82)
PTSD (PCL-5 ≥ 38)	2403	321 (13.4%)	1937	221 (11.4%)
Missing data		167 (6.5%)		163 (7.8%)
Dyspnoea-12^a^	2361	6.4 (8.2)	1892	5.7 (7.7)
Missing data		209 (8.1%)		208 (9.9%)
FACIT-Fatigue subscale score^a^	2326	34.6 (13.1)	1802	35.8 (12.7)
Missing data		244 (9.5%)		298 (14.2%)
BPI severity^a^	1847	13.2 (10.3)	1485	13.0 (10.0)
BPI interference^a^	1790	20.1 (19.5)	1435	19.5 (19.3)
Nottingham Extended ADL Scale^a^	2316	17.9 (5.0)	1780	18.4 (4.9)
**Physical performance**				
SPPB total score^a^	2342	9.8 (2.4)	1794	9.9 (2.2)
SPPB ≤ 10 (mobility disability)	2342	1196 (51.1%)	1794	860 (47.9%)
Missing data		228 (8.9%)		306 (14.6%)
ISWT distance (m)^a^	1975	423 (259)	1431	440 (253)
ISWT % predicted^a^	1399	57.1 (29.6)	1049	59.1 (27.9)
**Frailty and cognition**				
Rockwood CF score^b^	2285	3 [2–3]	1885	3 [2–3]
RCF ≥ 5	2285	135 (5.9%)		104 (5.5%)
Missing data		285 (11.1%)		215 (10.2%)
SARC-F total score^b^	2326	1 [0–3]	1808	1 [0–3]
Missing data		244 (9.5%)		292 (13.9%)
MoCA total score^a^	2100	25.6 (3.5)	1682	26.3 (3.4)
Corrected MoCA total score^a^	2100	25.9 (3.5)	1682	26.6 (3.3)
MoCA < 23	2100	321 (12.1%)	1682	199 (11.8%)
Corrected MoCA < 23	2100	279 (10.5%)	1682	178 (10.9%)
Missing data		470 (18.3%)		418 (19.9%)
**Lung physiology**				
FEV1 (L)^a^	1515	2.76 (0.80)	1081	2.81 (0.82)
Missing data		1055 (41.1%)		1019 (48.5%)
FEV1 % predicted^a^	1438	90.1 (18.5)	1051	91.7 (18.5)
Missing data		1132 (44.0%)		1049 (49.9%)
FEV1 % predicted < 80%	1438	389 (27.1%)	1051	257 (24.5%)
Missing data		1132 (44.0%)		1049 (49.9%)
FVC (L)^a^	1515	3.47 (1.02)	1081	3.56 (1.00)
Missing data		1055 (41.1%)		1019 (48.5%)
FVC % predicted^a^	1440	89.2 (18.6)	1049	91.1 (18.1)
Missing data		1130 (43.9%)		1051 (50.0%)
FVC % predicted < 80%	1440	427 (29.7%)	1049	260 (24.8%)
Missing data		1130 (43.9%)		1051 (50.0%)
FEV1/FVC^a^	1515	0.80 (0.15)	1079	0.79 (0.09)
Missing data		1055 (41.1%)		1021 (48.6%)
FEV1/FVC < 0.7	1515	163 (10.8%)	1079	118 (10.9%)
Missing data		1055 (41.1%)		1021 (48.6%)
TLCO mmol/KPa/min^a^	511	7.42 (2.33)	339	7.62 (2.19)
Missing data		2059 (80.1%)		1761 (83.9%)
TLCO % predicted^a^	499	91.6 (31.2)	336	94.7 (26.6)
Missing data		2071 (80.6%)		1764 (84.0%)
TLCO % predicted < 80%	499	175 (35.1%)	336	78 (23.2%)
Missing data		2071 (80.6%)		1764 (84.0%)
KCO mmol/KPa/min^a^	519	1.45 (0.29)	353	1.44 (0.27)
Missing data		2051 (79.8%)		1747 (83.2%)
KCO % predicted^a^	506	100.6 (18.6)	350	100.5 (17.5)
Missing data		2064 (80.3%)		1750 (83.3%)
KCO % predicted < 80%	506	45 (8.9%)	350	33 (9.3%)
Missing data		2064 (80.3%)		1750 (83.3%)
**Biochemical tests**				
BNP results (ng/L)^a^	152	98.9 (328.9)	59	82.5 (157.1)
Missing data		2418 (94.1%)		2041 (97.2%)
Pro-NT-BNP (ng/L)^a^	1439	150.6 (674.5)	1004	187.9 (848.4)
Missing data		1131 (44.0%)		1096 (52.2%)
BNP/Pro-NT-BNP above threshold	1591	107 (6.7%)	1063	93 (8.7%)
Missing data		979 (38.1%)		1037 (49.4%)
HbA1C % (DCCT/NGSP)^a^	1638	6.1 (1.2)	1289	6.2 (1.3)
Missing data		932 (36.3%)		811 (38.6%)
HbA1C ≥ 6.0	1638	579 (35.3%)	1289	463 (35.9%)
Missing data		932 (36.3%)		811 (38.6%)
eGFR (mL/min/1.73 m^2^)^a^	2105	76.6 (15.6)	1600	74.6 (16.4)
Missing data		465 (18.1%)		500 (23.8%)
eGFR < 60 (mL/min/1.73 m^2^)	2105	238 (11.3%)	1600	207 (12.9%)
Missing data		465 (18.1%)		500 (23.8%)
**Systemic inflammation**				
CRP (mg/L)^a^	2075	5.5 (11.3)	1636	5.1 (6.9)
Missing data		495 (19.3%)		464 (22.1%)
CRP > 5 mg/L	2075	502 (24.2%)	1636	393 (24.0%)
Missing data		495 (19.3%)		464 (22.1%)
CRP ≥ 10 mg/L	2075	231 (11.1%)	1636	174 (10.6%)
Missing data		495 (19.3%)		464 (22.1%)
Ferritin (µg/L)^a^	1832	143.7 (170.6)	1399	140.1 (189.4)
Missing data		738 (28.7%)		701 (33.4%)
Fibrinogen (g/L)^a^	1565	3.5 (0.9)	1310	3.5 (0.8)
Missing data		1005 (39.1%)		790 (37.6%)

Data are *n* (%) unless indicated. Missing data not included in %.

a Mean (SD).

b Median [IQR].

Threshold of BNP ≥ 100 ng/L or NT-BNP ≥ 400 ng/L. Corrected MoCA adjusted for level of education. See Supplementary Table SM1 (available as Supplementary data at *IJE* online) for further descriptions of variables.

ADL, activities of daily living; BNP, brain natriuretic peptide; BPI, Brief Pain Inventory Questionnaire; CF, clinical frailty; CFS, Clinical Frailty Scale; CRP, C-reactive protein; DCCT/NGSP, Diabetes Control and Complications Trial/National Glycohemoglobin Standardization Program; eGFR, estimated glomerular filtration rate; FACIT, Functional Assessment of Chronic Illness Therapy; FEV1, forced expiratory volume measured in 1 s; FVC, forced vital capacity; GAD7, Generalized Anxiety Disorder 7-item scale; HbA1C, glycated haemoglobin; ISWT, incremental shuttle walk test; KCO, carbon monoxide transfer coefficient; MoCA, Montreal Cognitive Assessment; NEADL, Nottingham Activities of Daily Living Questionnaire; NT-BNP, N-terminal BNP; PCL-5, Post-Traumatic Stress Disorder Checklist; PHQ-9, Patient Health Questionnaire-9; PROMs, patient-reported outcome measures; SPPB, short physical performance battery; TLCO, transfer capacity of the lung for carbon monoxide.

All assessments were performed as part of the two dedicated research visits except when relevant measures were already available from clinical follow-up appointments at the corresponding time points to reduce procedures burden and duplication.

All Tier 2 participants were invited to provide blood, urine, oral rinse and sputum samples for research purposes. Six different blood-sample tube types were used: plasma (EDTA, lithium heparin, citrate), serum, DNA and RNA (Supplementary Table S3, available as Supplementary data at *IJE* online). All samples were minimally processed at the local site before being shipped at intervals for longer-term storage at a central laboratory. This centralization of samples facilitated their use in multisite studies. Participants were asked to consent to use of their samples by other researchers, including commercial parties, both in the UK and abroad. Participants were given an option to decline their consent for genetic studies.

The participants’ consent to access healthcare records allowed access to and acquisition of clinically indicated images including chest X-ray and thoracic CT scans from certain participating sites, which were transferred to a national imaging database (National COVID-19 Chest Imaging Database) for analysis and secure storage (Supplementary Table S4, available as Supplementary data at *IJE* online).

Procedures for Tier 3 sub-studies were dependent on the specific criteria of the project, e.g. whole-body MRI imaging scans as part of the C-MORE sub-study (Supplementary Table S5, available as Supplementary data at *IJE* online), body composition measurements using dual energy X-ray analysis (DXA) imaging or further cognitive assessment using the Cognitron^12^ online test (Table 2).

### What have we achieved? Priority setting to identify 10 key research questions regarding the long-term sequelae of COVID-19

In order to ensure that the patient voice was central to the research undertaken using the PHOSP-COVID cohort, a joint patient and clinician priority setting exercise was undertaken between December 2020 and March 2021 to determine 10 priority research questions.^13^ The priority setting incorporated views from adults with self-reported long COVID, carers, clinicians, clinical researchers and charities including the Long Covid Support and Asthma + Lung UK. A modified version of the James Lind Alliance (JLA) priority setting partnerships process was used.^14^ A total of 119 initial questions were gathered prior to refining, rewording and grouping into a shorter list of 24 questions that was shared through an online prioritization survey receiving 882 responses. The final top 10 research questions were agreed at a dedicated prioritization workshop mediated by independent JLA facilitators and hosted via videoconference. The final top 10 research questions are listed in Supplementary Table S6 (available as Supplementary data at *IJE* online).

## What has it found?

### Significant burden of ongoing health impairment

Results from the first 1077 Tier 2 participants at 5 months post discharge highlighted that only 29% of participants felt fully recovered, 20% reported a new disability as assessed by using the Washington Group Short Set on Functioning (WG-SS) and 18% were no longer working.^11^ The 10 most-reported symptoms were: aching muscles, fatigue, physical slowing down, impaired sleep quality, joint pain or swelling, limb weakness, breathlessness, pain, short-term memory loss and a slowing-down in thinking. These findings were consistent with reported symptoms from smaller cohorts or cohorts of patients with a less severe initial illness.^15–17^ Around one in four of the cohort had clinically relevant symptoms of anxiety and depression, and nearly half of the participants had features of functional impairment measured using the Incremental Shuttle Walk Test and Short Physical Performance Battery at 5 months post discharge. There was also evidence of specific organ impairment: 35% had pre-diabetes or diabetes, 31% had impaired lung function, 17% had at least mild cognitive impairment, 13% had abnormal kidney function and 7% had raised brain natriuretic peptide (BNP). Further investigation of post-COVID residual lung abnormalities using clinical thoracic imaging at a median of 4 months post discharge revealed abnormalities affecting ≥10% of the lung were observed in 79.4% of a subset of 209 PHOSP-COVID participants.^18^ The prevalence of post-COVID residual lung abnormalities was estimated to be between 8.5% and 11.7%, and a proposed clinically applicable risk stratification suggested that 7.8% of the examined cohort had moderate to very-high risk of residual lung abnormalities post COVID hospitalization.

A striking finding was the lack of a clear association between the severity of the acute illness and the ongoing symptoms, mental and physical health impairments with the exception of pulmonary function tests and walking performance, which were worse in the group who received invasive mechanical ventilation.^11^

At 1 year after hospital discharge, there was very little improvement from 5 months in self-perceived recovery, ongoing symptoms, mental health, physical performance, and cognitive and organs impairment.^19^ The top 10 most prevalent symptoms were also similar to those at 5 months. Frailty and pre-frailty were present in more than two-thirds of participants at 1 year.^20^ A fall in the number of participants working at 1 year was seen, with 8.5% of those who were working before hospitalization no longer working and 34.6% of participants reporting that COVID-19 had resulted in a change in their occupation (Supplementary Table S7, available as Supplementary data at *IJE* online). Results from the complete Tier 2 cohort for the early and 1-year research visits are included in Tables 3 and 4.

**Table 4.** Health-related quality of life and disability among Tier 2 participants stratified by the research visits

	Available data (*n*)	Pre-COVID (*n*=2697)	Available data (*n*)	5 months (*n*=2570)	Available data (*n*)	1 year (*n*=2100)
EQ-5D-5L utility index^a^	2170	0.82 (0.23)	2113	0.71 (0.25)	1740	0.71 (0.25)
Missing data		527 (19.5%)		457 (17.8%)		360 (17.1%)
EQ-5D-5L utility index delta change^a^	–	–	1757	–0.11 (0.22)	1498	–0.11 (0.22)
Missing data				813 (31.6%)		602 (28.7%)
EQ-5D-5L VAS^a^	2095	79.5 (17.5)	2106	70.1 (20.0)	1731	70.4 (20.6)
Missing data		602 (22.3%)		464 (18.1%)		369 (17.6%)
EQ-5D-5L VAS delta change^a^	–	–	1697	– 9.9 (19.4)	1435	–9.8 (19.8)
Missing data				873 (33.9%)		665 (31.7%)
WG-SS-SCo	–	–	2208	532 (24.1%)	1793	389 (21.7%)
Missing data				362 (14.1%)		307 (14.6%)
WG-SS-SCo new disability	–	–	1659	317 (19.1%)	491	93 (18.9%)
Missing data				911 (35.5%)		1609 (76.6%)
PSQ Breathlessness^b^	2162	0 [0–2]	2193	4 [1–6]	1770	2 [0–5]
Missing data		535 (19.8%)		377 (14.7%)		330 (15.7%)
PSQ Cough^b^	2153	0 [0–1]	2184	1 [0–4]	1763	0 [0–2]
Missing data		544 (20.2%)		386 (15.0%)		337 (16.0%)
PSQ Fatigue^b^	2152	0 [0–2]	2183	5 [2–7]	1765	3 [1–6]
Missing data		545 (20.2%)		387 (15.1%)		335 (15.9%)
PSQ Poor Sleep^b^	2151	1 [0–4]	2177	4 [1–7]	1766	3 [0–6]
Missing data		546 (20.2%)		393 (15.3%)		334 (15.9%)
PSQ Pain^b^	2138	0 [0–3]	2169	3 [0–6]	1763	2 [0–5]
Missing data		559 (20.7%)		401 (15.6%)		337 (16.0%)

Data are *n* (%) unless indicated. Missing data not included in %.

a Mean (SD).

b Median [IQR].

See Supplementary Table SM1 (available as Supplementary data at *IJE* online) for further descriptions of variables.

EQ-5D-5L VAS, EuroQol five-level visual analogue scale 0–100; PSQ, Patient Symptoms Questionnaires; WG-SS-SCo, Washington Group Short Set of Functioning Severity Continuum.

### Risk factors for lack of recovery

The risk factors associated with lack of recovery at 1 year were: being female, being obese and having received invasive mechanical ventilation or other organ support during the acute illness.^19^ History of treatment with acute corticosteroids during the acute admission was not associated with any effect on patient-perceived recovery at 1 year despite the beneficial acute effects.^21^ Frailty was also positively associated with non-recovery and reduced health-related quality of life at 1 year following discharge.^20^

We identified risk factors for new or worse breathlessness post COVID at 5 months, including socio-economic deprivation, pre-existing depression/anxiety, female sex and longer hospital stay.^22^ Further analysis has also revealed disrupted sleep, present in 62% of the cohort, associated with dyspnoea, anxiety and muscle weakness, revealing an intriguing potential therapeutic intervention.^23^

### Recovery trajectory clusters

**Figure 2.**
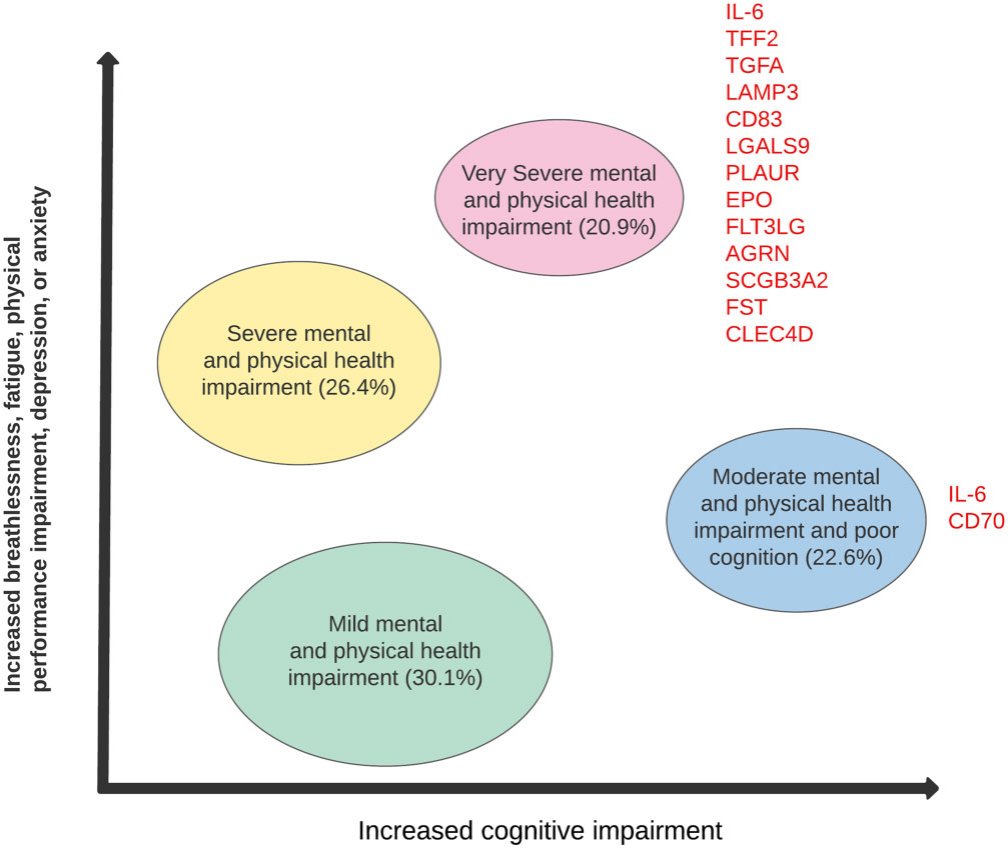
Illustration of the four cluster phenotypes of mental, cognitive and physical health impairments with associated inflammatory biomarkers. The figure shows the distribution of the four recovery cluster phenotypes and the list of identified proteins that were significantly differentially expressed (compared with the reference mild cluster) after FDR adjustment. FDR, false detection rate; IL-6, interleukin-6; TFF2, trefoil factor 2; TGFA, transforming growth factor α; LAMP3, lysosomal associated membrane protein 3; CD83, CD83 molecule; LGALS9, galectin-9; PLAUR, urokinase plasminogen activator surface receptor; EPO, erythropoietin; FLT3LG, FMS-related receptor tyrosine kinase 3 ligand; AGRN, agrin; SCGB3A2, secretoglobin family 3A member 2; FST, follistatin; CLEC4D, C-type lectin domain family 4 member D; CD70, CD70 molecule

We undertook unsupervised cluster modelling using validated objective measures of breathlessness, fatigue, anxiety, depression, post-traumatic stress disorder (PTSD), physical performance and cognitive impairments at 5 months and described four ‘recovery clusters’.^11^ The severity of most of the health impairments largely tracked together in the ‘very severe’, ‘severe’ and ‘mild’ clusters whereas the ‘moderate’ cluster was dominated by cognitive impairment (Figure 2). The more severe clusters were associated with female sex, higher body mass index (BMI), a higher number of symptoms, reduced physical function and elevated C-reactive protein levels. The ‘very severe’ recovery cluster was associated with fewer days/weeks containing continuous bouts of moderate-to-vigorous physical activity, longer total sleep time and higher variability in sleep timing.^24^ Although these are associations for which causal directions of effect have not been determined, these data highlight potential therapeutic targets.^25^

To investigate the inflammatory response further, levels of 296 inflammatory plasma proteins were measured at 5 months. Thirteen proteins including IL-6 were elevated in the ‘very severe’ and the ‘moderate with cognitive impairment’ clusters compared with the ‘mild cluster’ (Figure 2). These mediators of tissue damage and repair provide plausible biological mechanisms behind the symptoms and health impairments associated with severe long COVID.^19^

## What are the main strengths and weaknesses?

The large number of clinical variables collected, coupled with the biological research sampling, makes PHOSP-COVID one of the largest deeply phenotyped cohorts of hospitalized COVID-19 survivors in the world. Cross-sectional and longitudinal multi-omics markers are being measured in Tier 2 participants. These may uncover underlying mechanistic pathways implicated in long-COVID pathology and inform interventional trials. We have linked participants in PHOSP-COVID to the International Severe Acute Respiratory and emerging Infection Consortium (ISARIC) study data, where applicable.^26^ This provides additional information and linkage to samples taken during acute hospital admission. We are currently linking to other resources including vaccine data, viral strain data and electronic healthcare records, e.g. OpenSAFELY.

The multidimensional results generated by the PHOSP-COVID cohort are helping to shape and prioritize provision of clinical care at times when the national health services, both locally and globally, are under significant pressure after the pandemic.^27^ Setting priority research questions and identifying risk groups will focus the efforts of both clinical and academic institutions at managing the large volume of patients with long COVID.^13^^,^^28^

The study was designed as a cohort, with the study population being defined as COVID-19 hospitalized survivors with a range of outcomes captured enabling nested case–control analyses. As such, no external comparator groups (i.e. non-hospitalized COVID-19 survivors, individuals hospitalized with other viral infections) were recruited to the study. However, this has been partially mitigated by using external cohorts or healthy controls to examine certain hypotheses.^29^

As participants were prospectively recruited following discharge from hospital, data pertaining to pre-COVID-19 health status were only available from healthcare records or by participant recall, introducing the potential for recall bias. There is also unavoidable selection bias as some of the participants might have accepted the invitation to the study due to the severity of their ongoing symptoms. This is particularly relevant to Tier 2 participants, who were younger, more ethnically diverse, less comorbid and required more respiratory support compared with the participants included in the ISARIC consortium outputs, which are likely more representative of the overall hospitalized population in the UK.^30^ However, the linkage to ISARIC and other public databases may help to quantify and partially mitigate this bias.

As the PHOSP-COVID cohort included participants from 83 different sites and due to the pressure associated with providing clinical and academic services during the heights of the pandemic, there were considerable variations in the availability of collected data across these multiple sites. However, the large number of recruited participants still makes the PHOSP-COVID one of the largest multicentre cohorts globally.

As recruitment began in August 2020, the cohort represents mainly patients who were admitted to hospital during the first year of the pandemic and so mostly preceded the emergence of the Delta and Omicron SARS-CoV2 variants, and the wide use of in-hospital acute therapies. In addition, as vaccination in the UK did not begin until late 2020, a large proportion of the cohort were vaccine naïve at initial hospital admission and at the 5-month follow-up.

## Can I get hold of the data? Where can I find out more?

**Figure 3.**
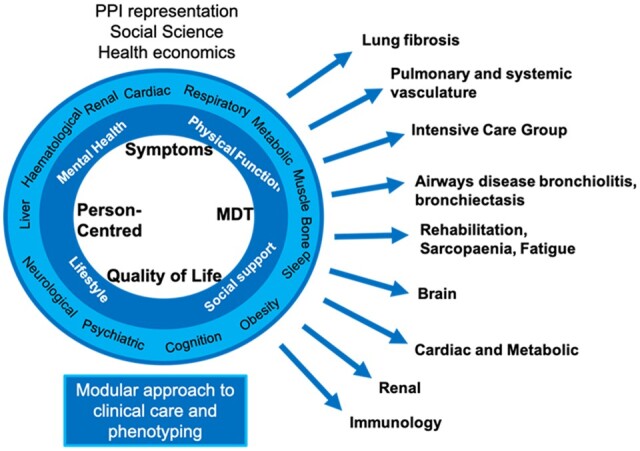
Modular approach to the clinical care and phenotyping with the different working groups of the Post-Hospitalisation COVID-19 (PHOSP-COVID) consortium. MDT, multidisciplinary team; PPI, patient and public involvement

The PHOSP-COVID study website (https://www.phosp.org) contains an overview of the study, resources, information about people involved and publications. Research activity using the study is organized across a series of working groups (Figure 3). These were established at the outset of the study to coordinate research, minimize duplication of efforts and facilitate communication across research and clinical specialties. Researchers interested in undertaking research using PHOSP-COVID are encouraged to contact the relevant working group leads (https://www.phosp.org/working-group/) in the first instance. The data are currently held in the Outbreak Data Analysis Platform (ODAP, https://odap.ac.uk/). Researchers seeking to access these data are directed to https://www.phosp.org/resource/ for information and forms. Correspondence to be directed to Dr Rachael A Evans, the Co-Principal Investigator of PHOSP-COVID study, at phosp@leicester.ac.uk.

## Notes

### PHOSP-COVID collaborative group

#### Core management group

Chief Investigator: CE Brightling. Members: RA Evans (Lead Co-I), LV Wain (Lead Co-I), JD Chalmers, VC Harris, LP Ho, A Horsley, M Marks, K Poinasamy, B Raman, A Shikotra, A Singapuri

#### PHOSP-COVID Study Central Coordinating Team

CE Brightling (Chief Investigator), RA Evans (Lead Co-I), LV Wain (Lead Co-I), R Dowling, C Edwardson, O Elneima, S Finney, NJ Greening, B Hargadon, VC Harris, L Houchen-Wolloff, OC Leavy, HJC McAuley, C Overton, T Plekhanova, RM Saunders, M Sereno, A Singapuri, A Shikotra, C Taylor, S Terry, C Tong, B Zhao

#### Steering Committee

Co-chairs: D Lomas, E Sapey; Institution representatives: C Berry, CE Bolton, N Brunskill, ER Chilvers, R Djukanovic, Y Ellis, D Forton, N French, J George, NA Hanley, N Hart, L McGarvey, N Maskell, H McShane, M Parkes, D Peckham, P Pfeffer, A Sayer, A Sheikh, AAR Thompson, N Williams and core management group representation

#### Executive Board

Chair: CE Brightling; representation from the core management group, each working group and platforms

### Platforms

#### Bioresource

W Greenhalf (Co-Lead), MG Semple (Co-Lead), M Ashworth, HE Hardwick, L Lavelle-Langham, W Reynolds, M Sereno, RM Saunders, A Singapuri, V Shaw, A Shikotra, B Vinson, LV Wain

#### Data hub

AB Docherty (Co-Lead), EM Harrison (Co-Lead), A Sheikh (Co-Lead), JK Baillie, CE Brightling, L Daines, R Free, RA Evans, S Kerr, OC Leavy, NI Lone, HJC McAuley, R Pius, JK Quint, M Richardson, M Sereno, M Thorpe, LV Wain

#### Imaging alliance

M Halling-Brown (Co-Lead), F Gleeson (Co-Lead), J Jacob (Co-Lead), S Neubauer (Co-Lead), B Raman (Co-Lead), S Siddiqui (Co-Lead), JM Wild (Co-Lead), S Aslani, G Baxter, M Beggs, C Bloomfield, MP Cassar, A Chiribiri, E Cox, DJ Cuthbertson, M Halling-Brown, VM Ferreira, L Finnigan, S Francis, P Jezzard, GJ Kemp, H Lamlum, E Lukaschuk, C Manisty, GP McCann, C McCracken, K McGlynn, R Menke, CA Miller, AJ Moss, TE Nichols, C Nikolaidou, C O’Brien, G Ogbole, B Rangelov, DP O’Regan, A Pakzad, S Piechnik, S Plein, I Propescu, AA Samat, L Saunders, ZB Sanders, R Steeds, T Treibel, EM Tunnicliffe, M Webster, J Willoughby, J Weir McCall, C Xie, M Xu

#### Omics

LV Wain (Co-Lead), JK Baillie (Co-Lead), H Baxendale, CE Brightling, M Brown, JD Chalmers, RA Evans, B Gooptu, W Greenhalf, HE Hardwick, RG Jenkins, D Jones, I Koychev, C Langenberg, A Lawrie, PL Molyneaux, A Shikotra, J Pearl, M Ralser, N Sattar, RM Saunders, JT Scott, T Shaw, D Thomas, D Wilkinson

### Working groups

#### Airways

LG Heaney (Co-Lead), A De Soyza (Co-Lead), D Adeloye, CE Brightling, JS Brown, J Busby, JD Chalmers, C Echevarria, L Daines, O Elneima, RA Evans, JR Hurst, P Novotny, C Nicolaou, P Pfeffer, K Poinasamy, JK Quint, I Rudan, E Sapey, M Shankar-Hari, A Sheikh, S Siddiqui, S Walker, B Zheng

#### Brain

JR Geddes (Lead), M Hotopf (Co-Lead), K Abel, R Ahmed, L Allan, C Armour, D Baguley, D Baldwin, C Ballard, K Bhui, G Breen, K Breeze, M Broome, T Brugha, E Bullmore, D Burn, F Callard, J Cavanagh, T Chalder, D Clark, A David, B Deakin, H Dobson, B Elliott, J Evans, RA Evans, R Francis, E Guthrie, P Harrison, M Henderson, A Hosseini, N Huneke, M Husain, T Jackson, I Jones, T Kabir, P Kitterick, A Korszun, I Koychev, J Kwan, A Lingford-Hughes, P Mansoori, H McAllister-Williams, K McIvor, B Michael, L Milligan, R Morriss, E Mukaetova-Ladinska, K Munro, A Nevado-Holgado, T Nicholson, C Nicolaou, S Paddick, C Pariante, J Pimm, K Saunders, M Sharpe, G Simons, JP Taylor, R Upthegrove, S Wessely

#### Cardiac

GP McCann (Lead), S Amoils, C Antoniades, A Banerjee, A Bularga, C Berry, P Chowienczyk, JP Greenwood, AD Hughes, K Khunti, C Lawson, NL Mills, AJ Moss, S Neubauer, B Raman, AN Sattar, CL Sudlow, M Toshner,

#### Immunology

PJM Openshaw (Lead), D Altmann, JK Baillie, R Batterham, H Baxendale, N Bishop, CE Brightling, PC Calder, RA Evans, JL Heeney, T Hussell, P Klenerman, JM Lord, P Moss, SL Rowland-Jones, W Schwaeble, MG Semple, RS Thwaites, L Turtle, LV Wain, S Walmsley, D Wraith

#### Intensive care

MJ Rowland (Lead), A Rostron (Co-Lead), JK Baillie, B Connolly, AB Docherty, NI Lone, DF McAuley, D Parekh, A Rostron, J Simpson, C Summers

#### Lung fibrosis

RG Jenkins (Co-Lead), J Porter (Co-Lead), RJ Allen, R Aul, JK Baillie, S Barratt, P Beirne, J Blaikley, RC Chambers, N Chaudhuri, C Coleman, E Denneny, L Fabbri, PM George, M Gibbons, F Gleeson, B Gooptu, B Guillen Guio, I Hall, NA Hanley, LP Ho, E Hufton, J Jacob, I Jarrold, G Jenkins, S Johnson, MG Jones, S Jones, F Khan, P Mehta, J Mitchell, PL Molyneaux, JE Pearl, K Piper Hanley, K Poinasamy, J Quint, D Parekh, P Rivera-Ortega, LC Saunders, MG Semple, J Simpson, D Smith, M Spears, LG Spencer, S Stanel, I Stewart, AAR Thompson, D Thickett, R Thwaites, LV Wain, S Walker, S Walsh, JM Wild, DG Wootton, L Wright

#### Metabolic

S Heller (Co-Lead), MJ Davies (Co-Lead), H Atkins, S Bain, J Dennis, K Ismail, D Johnston, P Kar, K Khunti, C Langenberg, P McArdle, A McGovern, T Peto, J Petrie, E Robertson, N Sattar, K Shah, J Valabhji, B Young

#### Pulmonary and systematic vasculature

LS Howard (Co-Lead), Mark Toshner (Co-Lead), C Berry, P Chowienczyk, A Lawrie, OC Leavy, J Mitchell, J Newman, L Price, J Quint, A Reddy, J Rossdale, N Sattar, C Sudlow, AAR Thompson, JM Wild, M Wilkins

#### Rehabilitation, sarcopenia and fatigue

SJ Singh (Co-Lead), WD-C Man (Co-Lead), JM Lord (Co-Lead), NJ Greening (Co-Lead), T Chalder (Co-Lead), JT Scott (Co-Lead), N Armstrong, E Baldry, M Baldwin, N Basu, M Beadsworth, L Bishop, CE Bolton, A Briggs, M Buch, G Carson, J Cavanagh, H Chinoy, C Dawson, E Daynes, S Defres, RA Evans, L Gardiner, P Greenhaff, S Greenwood, M Harvie, L Houchen-Wolloff, M Husain, S MacDonald, A McArdle, HJC McAuley, A McMahon, M McNarry, G Mills, C Nolan, K O’Donnell, D Parekh, Pimm, J Sargent, L Sigfrid, M Steiner, D Stensel, AL Tan, I Vogiatzis, J Whitney, D Wilkinson, D Wilson, M Witham, DG Wootton, T Yates

#### Renal

D Thomas (Lead), N Brunskill (Co-Lead), S Francis (Co-Lead), S Greenwood (Co-Lead), C Laing (Co-Lead), K Bramham, P Chowdhury, A Frankel, L Lightstone, S McAdoo, K McCafferty, M Ostermann, N Selby, C Sharpe, M Willicombe

#### Patient Public Engagement Group

L Houchen-Wolloff (Lead), J Bunker, R Gill, C Hastie, R Nathu, N Rogers, N Smith

### Local clinical centre PHOSP-COVID trial staff

(listed in alphabetical order)

#### Airedale NHS Foundation Trust

A Shaw (PI), L Armstrong, B Hairsine, H Henson, C Kurasz, L Shenton

#### Aneurin Bevan University Health Board

S Fairbairn (PI), A Dell, N Hawkings, J Haworth, M Hoare, A Lucey, V Lewis, G Mallison, H Nassa, C Pennington, A Price, C Price, A Storrie, G Willis, S Young

#### Barts Health NHS Trust & Queen Mary University of London

P Pfeffer (PI), K Chong-James, C David, WY James, C Manisty, A Martineau, O Zongo

#### Barnsley Hospital NHS Foundation Trust

A Sanderson (PI)

#### Belfast Health and Social Care Trust & Queen's University Belfast

LG Heaney (PI), C Armour, V Brown, T Craig, S Drain, B King, N Magee, D McAulay, E Major, L McGarvey, J McGinness, R Stone

#### Betsi Cadwaladr University Health Board

A Haggar (PI), A Bolger, F Davies, J Lewis, A Lloyd, R Manley, E McIvor, D Menzies, K Roberts, W Saxon, D Southern, C Subbe, V Whitehead

#### Borders General Hospital, NHS Borders

H El-Taweel (PI), J Dawson, L Robinson

#### Bradford Teaching Hospitals NHS Foundation Trust

D Saralaya (PI), L Brear, K Regan, K Storton

#### Cambridge University Hospitals NHS Foundation Trust, NIHR Cambridge Clinical Research Facility & University of Cambridge

J Fuld (PI), A Bermperi, I Cruz, K Dempsey, A Elmer, H Jones, S Jose, S Marciniak, M Parkes, C Ribeiro, J Taylor, M Toshner, L Watson, J Weir McCall, J Worsley

#### Cardiff and Vale University Health Board

R Sabit (PI), L Broad, A Buttress, T Evans, M Haynes, L Jones, L Knibbs, A McQueen, C Oliver, K Paradowski, J Williams

#### Chesterfield Royal Hospital NHS Trust

E Harris (PI), C Sampson

#### Cwm Taf Morgannwg University Health Board

C Lynch (PI), E Davies, C Evenden, A Hancock, K Hancock, M Rees, L Roche, N Stroud, T Thomas-Woods

#### East Cheshire NHS Trust

M Babores (PI), J Bradley-Potts, M Holland, N Keenan, S Shashaa, H Wassall

#### East Kent Hospitals University NHS Foundation Trust

E Beranova (PI), H Weston (PI), T Cosier, L Austin, J Deery, T Hazelton, C Price, H Ramos, R Solly, S Turney

#### Gateshead NHS Trust

L Pearce (PI), W McCormick, S Pugmire, W Stoker, A Wilson

#### Guy’s and St Thomas’ NHS Foundation Trust

N Hart (PI), LA Aguilar Jimenez, G Arbane, S Betts, K Bisnauthsing, A Dewar, P Chowdhury, A Chiribiri, A Dewar, G Kaltsakas, H Kerslake, MM Magtoto, P Marino, LM Martinez, C O'Brien, M Ostermann, J Rossdale, TS Solano, E Wynn

#### Hampshire Hospitals NHS Foundation Trust

N Williams (PI), W Storrar (PI), M Alvarez Corral, A Arias, E Bevan, D Griffin, J Martin, J Owen, S Payne, A Prabhu, A Reed, C Wrey Brown

#### Harrogate and District NHD Foundation Trust

C Lawson (PI), T Burdett, J Featherstone, A Layton, C Mills, L Stephenson

#### Health and Care Research Wales

Y Ellis

#### Hull University Teaching Hospitals NHS Trust & University of Hull

N Easom (PI), P Atkin, K Brindle, MG Crooks, K Drury, R Flockton, L Holdsworth, A Richards, DL Sykes, S Thackray-Nocera, C Wright

#### Hywel Dda University Health Board

KE Lewis (PI), A Mohamed (PI), G Ross (PI), S Coetzee, K Davies, R Hughes, R Loosley, L O’Brien, Z Omar, H McGuinness, E Perkins, J Phipps, A Taylor, H Tench, R Wolf-Roberts

#### Imperial College Healthcare NHS Trust & Imperial College London

LS Howard (PI), O Kon (PI), DC Thomas (PI), S Anifowose, L Burden, E Calvelo, B Card, C Carr, ER Chilvers, D Copeland, P Cullinan, P Daly, L Evison, T Fayzan, H Gordon, S Haq, RG Jenkins, C King, K March, M Mariveles, L McLeavey, N Mohamed, S Moriera, U Munawar, J Nunag, U Nwanguma, L Orriss-Dib, DP O'Regan, A Ross, M Roy, E Russell, K Samuel, J Schronce, N Simpson, L Tarusan, C Wood, N Yasmin

#### Kettering General Hospital NHS Trust

R Reddy (PI), A-M Guerdette, M Hewitt, K Warwick, S White

#### King’s College Hospital NHS Foundation Trust & Kings College London

AM Shah (PI), CJ Jolley (PI), O Adeyemi, R Adrego, H Assefa-Kebede, J Breeze, M Brown, S Byrne, T Chalder, A Chiribiri, P Dulawan, N Hart, A Hayday, A Hoare, A Knighton, M Malim, C O'Brien, S Patale, I Peralta, N Powell, A Ramos, K Shevket, F Speranza, A Te

#### Leeds Teaching Hospitals & University of Leeds

P Beirne (PI), A Ashworth, J Clarke, C Coupland, M Dalton, E Wade, C Favager, J Greenwood, J Glossop, L Hall, T Hardy, A Humphries, J Murira, D Peckham, S Plein, J Rangeley, G Saalmink, AL Tan, B Whittam, N Window, J Woods,

#### Lewisham & Greenwich NHS Trust

G Coakley (PI)

#### Liverpool University Hospitals NHS Foundation Trust & University of Liverpool

DG Wootton (PI), L Turtle (PI), L Allerton, AM All, M Beadsworth, A Berridge, J Brown, S Cooper, A Cross, DJ Cuthbertson, S Defres, SL Dobson, J Earley, N French, W Greenhalf, HE Hardwick, K Hainey, J Hawkes, V Highett, S Kaprowska, GJ Kemp, AL Key, S Koprowska, L Lavelle-Langham, N Lewis-Burke, G Madzamba, F Malein, S Marsh, C Mears, L Melling, MJ Noonan, L Poll, J Pratt, E Richardson, A Rowe, MG Semple, V Shaw, KA Tripp, B Vinson, LO Wajero, SA Williams-Howard, J Wyles

#### London North West University Healthcare NHS Trust

SN Diwanji (PI), P Papineni (PI), S Gurram, S Quaid, GF Tiongson, E Watson

#### London School of Hygiene & Tropical Medicine (LSHTM)

M Marks, A Briggs

#### Manchester University NHS Foundation Trust & University of Manchester

B Al-Sheklly (PI), A Horsley (PI), C Avram, J Blaikley, M Buch, N Choudhury, D Faluyi, T Felton, T Gorsuch, NA Hanley, T Hussell, Z Kausar, CA Miller, N Odell, R Osbourne, K Piper Hanley, K Radhakrishnan, S Stockdale

#### Newcastle upon Tyne Hospitals NHS Foundation Trust & University of Newcastle

A De Soyza (PI), C Echevarria (PI), A Ayoub, J Brown, G Burns, G Davies, H Fisher, C Francis, A Greenhalgh, P Hogarth, J Hughes, K Jiwa, G Jones, G MacGowan, D Price, A Sayer, J Simpson, H Tedd, S Thomas, S West, M Witham, S Wright, A Young

#### NHS Dumfries and Galloway

MJ McMahon (PI), P Neill

#### NHS Greater Glasgow and Clyde Health Board & University of Glasgow

D Anderson (PI), H Bayes (PI), C Berry (PI), D Grieve (PI), IB McInnes (PI), N Basu, A Brown, A Dougherty, K Fallon, L Gilmour, K Mangion, A Morrow, K Scott, R Sykes, R Touyz

#### NHS Highland

EK Sage (PI), F Barrett, A Donaldson

#### NHS Lanarkshire

M Patel (PI), D Bell, A Brown, M Brown, R Hamil, K Leitch, L Macliver, J Quigley, A Smith, B Welsh

#### NHS Lothian & University of Edinburgh

G Choudhury (PI), JK Baillie, S Clohisey, A Deans, AB Docherty, J Furniss, EM Harrison, S Kelly, NI Lone, DE Newby, A Sheikh

#### NHS Tayside & University of Dundee

JD Chalmers (PI), D Connell, A Elliott, C Deas, J George, S Mohammed, J Rowland, AR Solstice, D Sutherland, CJ Tee

#### NIHR Office for Clinical Research Infrastructure

K Holmes

#### North Bristol NHS Trust & University of Bristol

N Maskell (PI), D Arnold, S Barrett, H Adamali, A Dipper, S Dunn, A Morley, L Morrison, L Stadon, S Waterson, H Welch

#### North Middlesex Hospital NHS Trust

B Jayaraman (PI), T Light

#### Nottingham University Hospitals NHS Trust & University of Nottingham

CE Bolton (PI), P Almeida, J Bonnington, M Chrystal, E Cox, C Dupont, S Francis, P Greenhaff, A Gupta, L Howard, W Jang, S Linford, L Matthews, R Needham, A Nikolaidis, S Prosper, K Shaw, AK Thomas

#### Oxford University Hospitals NHS Foundation Trust & University of Oxford

LP Ho (PI), NM Rahman (PI), M Ainsworth, A Alamoudi, M Beggs, A Bates, A Bloss, A Burns, P Carter, M Cassar, KM Channon, J Chen, F Conneh, T Dong, RI Evans, E Fraser, X Fu, JR Geddes, F Gleeson, P Harrison, M Havinden-Williams, P Jezzard, N Kanellakis, I Koychev, P Kurupati, X Li, E Lukaschuk, K McGlynn, H McShane, C Megson, K Motohashi, S Neubauer, D Nicoll, G Ogg, E Pacpaco, M Pavlides, Y Peng, N Petousi, J Propescu, N Rahman, B Raman, MJ Rowland, K Saunders, M Sharpe, N Talbot, E Tunnicliffe

#### Patient Public Involvement Leads

Asthma UK and British Lung Foundation Partnership—K Poinasamy, S Walker

#### Royal Brompton and Harefield Clinical Group, Guy’s and St Thomas’ NHS Foundation Trust

WD-C Man (PI), B Patel (PI), RE Barker, D Cristiano, N Dormand, M Gummadi, S Kon, K Liyanage, CM Nolan, S Patel, O Polgar, P Shah, SJ Singh, JA Walsh

#### Royal Free London NHS Foundation Trust

JR Hurst (PI), H Jarvis (PI), S Mandal (PI), S Ahmad, S Brill, L Lim, D Matila, O Olaosebikan, C Singh

#### Royal Papworth Hospital NHS Foundation Trust

M Toshner (PI), H Baxendale, L Garner, C Johnson, J Mackie, A Michael, J Pack, K Paques, H Parfrey, J Parmar

#### Royal Surrey NHS Foundation Trust

M Halling-Brown

#### Salford Royal NHS Foundation Trust

N Diar Bakerly (PI), P Dark, D Evans, E Hardy, A Harvey, D Holgate, S Knight, N Mairs, N Majeed, L McMorrow, J Oxton, J Pendlebury, C Summersgill, R Ugwuoke, S Whittaker

#### Salisbury NHS Foundation Trust

W Matimba-Mupaya (PI), S Strong-Sheldrake

#### Sheffield Teaching NHS Foundation Trust & University of Sheffield

SL Rowland-Jones (PI), AAR Thompson (Co PI), J Bagshaw, M Begum, K Birchall, R Butcher, H Carborn, F Chan, K Chapman, Y Cheng, L Chetham, C Clark, Z Coburn, J Cole, M Dixon, A Fairman, J Finnigan, L Finnigan, H Foot, D Foote, A Ford, R Gregory, K Harrington, L Haslam, L Hesselden, J Hockridge, A Holbourn, B Holroyd-Hind, L Holt, A Howell, E Hurditch, F Ilyas, C Jarman, A Lawrie, E Lee, J-H Lee, R Lenagh, A Lye, I Macharia, M Marshall, A Mbuyisa, J McNeill, S Megson, J Meiring, L Milner, S Misra, H Newell, T Newman, C Norman, L Nwafor, D Pattenadk, M Plowright, J Porter, P Ravencroft, C Roddis, J Rodger, P Saunders, J Sidebottom, J Smith, L Smith, N Steele, G Stephens, R Stimpson, B Thamu, N Tinker, K Turner, H Turton, P Wade, S Walker, J Watson, JM Wild, I Wilson, A Zawia

#### St George’s University Hospitals NHS Foundation Trust

R Aul (PI), M Ali, A Dunleavy (PI), D Forton, N Msimanga, M Mencias, T Samakomva, S Siddique, J Teixeira, V Tavoukjian

#### Sherwood Forest Hospitals NHS Foundation Trust

J Hutchinson (PI), L Allsop, K Bennett, P Buckley, M Flynn, M Gill, C Goodwin, M Greatorex, H Gregory, C Heeley, L Holloway, M Holmes, J Kirk, W Lovegrove, TA Sewell, S Shelton, D Sissons, K Slack, S Smith, D Sowter, S Turner, V Whitworth, I Wynter

#### Shropshire Community Health NHS Trust

L Warburton (PI), S Painter, J Tomlinson

#### Somerset NHS Foundation Trust

C Vickers (PI), T Wainwright, D Redwood, J Tilley, S Palmer

#### South London and Maudsley NHS Foundation Trust & Kings College London

G Breen, M Hotopf

#### Swansea Bay University Health Board

GA Davies (PI), L Connor, A Cook, T Rees, F Thaivalappil, C Thomas

#### Swansea University & Swansea Welsh Network

K Lewis, N Williams

#### Tameside and Glossop Integrated Care NHS Foundation

A Butt (PI), M Coulding, H Jones, S Kilroy, J McCormick, J McIntosh, H Savill, V Turner, J Vere

#### The Great Western Hospital Foundation Trust

E Fraile (PI), J Ugoji

#### The Hillingdon Hospitals NHS Foundation Trust

SS Kon (PI), H Lota, G Landers, M Nasseri, S Portukhay

#### The Rotherham NHS Foundation Trust

A Hormis (PI), A Daniels, J Ingham, L Zeidan

#### United Lincolnshire Hospitals NHS Trust

M Chablani (PI), L Osborne

#### University College London Hospital & University College London

M Marks (PI), JS Brown (PI), N Ahwireng, B Bang, D Basire, RC Chambers, A Checkley, R Evans, M Heightman, T Hillman, J Hurst, J Jacob, S Janes, R Jastrub, M Lipman, S Logan, D Lomas, M Merida Morillas, A Pakzad, H Plant, JC Porter, K Roy, E Wall, B Williams, M Xu

#### University Hospital Birmingham NHS Foundation Trust & University of Birmingham

D Parekh (PI), N Ahmad Haider, C Atkin, R Baggott, M Bates, A Botkai, A Casey, B Cooper, J Dasgin, K Draxlbauer, N Gautam, J Hazeldine, T Hiwot, S Holden, K Isaacs, T Jackson, S Johnson, V Kamwa, D Lewis, JM Lord, S Madathil, C McGhee, K Mcgee, A Neal, A Newton Cox, J Nyaboko, D Parekh, Z Peterkin, H Qureshi, B Rangelov, L Ratcliffe, E Sapey, J Short, T Soulsby, R Steeds, J Stockley, Z Suleiman, T Thompson, M Ventura, S Walder, C Welch, D Wilson, S Yasmin, KP Yip

#### University Hospital Southampton NHS Foundation Trust & University of Southampton

MG Jones (PI), C Childs, R Djukanovic, S Fletcher, M Harvey, E Marouzet, B Marshall, R Samuel, T Sass, T Wallis, H Wheeler

#### University Hospitals of Derby and Burton

P Beckett (PI) C Dickens, U Nanda

#### University Hospitals of Leicester NHS Trust & University of Leicester

CE Brightling (CI), RA Evans (PI), M Aljaroof, N Armstrong, H Arnold, H Aung, M Bakali, M Bakau, M Baldwin, M Bingham, M Bourne, C Bourne, N Brunskill, P Cairns, L Carr, A Charalambou, C Christie, MJ Davies, S Diver, S Edwards, C Edwardson, O Elneima, H Evans, J Finch, S Glover, N Goodman, B Gooptu, NJ Greening, K Hadley, P Haldar, B Hargadon, VC Harris, L Houchen-Wolloff, W Ibrahim, L Ingram, K Khunti, A Lea, D Lee, GP McCann, HJC McAuley, P McCourt, T Mcnally, G Mills, A Moss, W Monteiro, M Pareek, S Parker, A Rowland, A Prickett, IN Qureshi, RJ Russell, N Samani, M Sereno, M Sharma, A Shikotra, S Siddiqui, A Singapuri, SJ Singh, J Skeemer, M Soares, E Stringer, T Thornton, M Tobin, E Turner, LV Wain, TJC Ward, F Woodhead, J Wormleighton, T Yates, A Yousuf

#### Whittington Health NHS

R Dharmagunawardena (PI), E Bright, P Crisp, M Stern

#### Wirral University Teaching Hospital

A Wight (PI), L Bailey, A Reddington

#### Wrightington Wigan and Leigh NHS trust

A Ashish (PI), J Cooper, E Robinson

#### Yeovil District Hospital NHS Foundation Trust

A Broadley (PI)

#### York & Scarborough NHS Foundation Trust

K Howard (PI), L Barman, C Brookes, K Elliott. L Griffiths, Z Guy, D Ionita, H Redfearn, C Sarginson, A Turnbull

## Ethics approval

The study was approved by the Leeds West Research Ethics Committee (20/YH/0225) and is registered on the ISRCTN Registry (ISRCTN10980107).

## Supplementary Material

dyad165_Supplementary_Data

## Data Availability

See ‘Can I get hold of the data?’ above.
