# Supplementary material for: Cohort Profile: Post-Hospitalisation COVID-19 (PHOSP-COVID) study
Source: Int J Epidemiol. 2023 Dec 18;53(1):dyad165. doi: 10.1093/ije/dyad165 (PMC10859139; doi:10.1093/ije/dyad165)
Supplement: dyad165_Supplementary_Data [file dyad165_supplementary_data.zip › ije-2023-05-0521-File006.docx]

**Supplementary file**

Cohort Profile: Post-hospitalisation COVID-19 study (PHOSP-COVID)

*PHOSP-COVID Collaborative Group*

Contents

[Supplementary Methods 2](#_Toc142791654)

[Supplementary Data – Results 4](#_Toc142791655)

[References 8](#_Toc142791656)

# Supplementary Methods

**Table SM1. Methods and thresholds for processing of variables and outcome measures presented in this analysis.**

|  | **Method** |
| --- | --- |
| **Tables 1 & S2** |  |
| Indices of Multiple Deprivation (IMD) | Obtained using postcode^1^. Different modalities used for the devolved nations. |
| Comorbidities | A pre-existing comorbidity was considered absent if not indicated by a ‘yes’ on the case report form. |
| Admission duration | Calculated using the hospital discharge date and the earliest admission date to the same or different hospital for the participant’s COVID-19 episode. |
| WHO clinical progression scale | WHO classes are as follows: 3–4=no continuous supplemental oxygen needed; 5=continuous supplemental oxygen only; 6=continuous or bi-level positive airway pressure ventilation or high-flow nasal oxygen; and 7–9=invasive mechanical ventilation or other organ support e.g. vasopressors, dialysis or Extracorporeal membrane oxygenation (ECMO) ^2^. |
| **Table 3** |  |
| Generalised Anxiety Disorder Questionnaire (GAD-7) (Anxiety) | The Generalised Anxiety Disorder (GAD-7) questionnaire is a patient reported outcome measure consisting of 7 questions with total scores ranging from 0 to 21. We used a GAD7 threshold score of > 8 to suggest at least mild-moderate anxiety.^3^ |
| Patient Health Questionnaire (PHQ-9) (Depression) | The Patient Health Questionnaire (PHQ-9) is a patient reported outcome measure consisting of 9 questions with total scores ranging from 0 to 27. We used a PHQ-9 threshold score of ≥10 to suggest at least moderate depression.^4^ |
| Post-Traumatic Stress Disorder Checklist for DSM V (PCL-5) Questionnaire | The Post-Traumatic Stress Disorder Checklist for DSM V (PCL-5) questionnaire is a patient reported outcome measure consisting of 20 questions assessing evidence of post-traumatic stress disorder according to the DSM V criteria. Total scores range from 0-80. We used a PCL-5 threshold score of ≥38 suggestive of a provisional diagnosis of post-traumatic stress disorder.^5,6^ |
| Dyspnoea-12 | The Dyspnoea-12 questionnaire is a patient reported outcome measure consisting of 12 questions assessing breathlessness severity incorporating both “physical” and “affective” aspects^7^. Scores range from 0 to 36 with higher scores corresponding to greater severity of breathlessness. |
| FACIT fatigue subscale score (FACIT) | The Functional Assessment of Chronic Illness Therapy – Fatigue (FACIT-Fatigue) scale is a patient reported outcome measure consisting of 13 questions to assess self-reported fatigue and its impact on daily activities and function.^8^ Total scores range from 0-52, with lower scores corresponding to an increased burden of fatigue.^9^ |
| Brief Pain Inventory (BPI) severity and interference | The Brief Pain Inventory (BPI) is a patient reported outcome questionnaire consisting of 15 questions across domains of pain severity and pain interference. We have reported the BPI Severity score as the mean score from the 4 severity questions each with a range 0 – 10 anchored at 0 = “No Pain” and 10 = “Pain as bad as you can imagine”.^10,11^ |
| Short Physical Performance Battery (SPPB) | The Short Physical Performance Battery (SPPB) test is a researcher administered assessment of physical performance and frailty. It comprises 3 components; balance, gait speed and sit to stand tests. Tests were completed according to recommended standards and training was provided to site staff by the central study team via a recorded demonstration video. SPPB total scores range from 0-12. We have reported a total SPPB score of ≤10 suggestive of underlying frailty.^12-14^ |
| Incremental Shuttle Walk Test (ISWT) | The Incremental Shuttle Walk Test (ISWT) is a researcher administered assessment of maximal physical performance and was performed according to standardised instructions with two attempts performed by participants on the same day with a 20 minutes rest between them.^15^ Training was provided to site staff by the central study team via a recorded demonstration video. The best effort was reported in metres and the percent predicted value was calculated using the following reference formula accounting for gender, age and BMI.^16^ (ISWT predicted = 1449·701 − (11·735 × age) + (241·897 × gender) − (5·686 × BMI), where male gender = 1 and female gender = 0) |
| Rockwood Clinical Frailty Scale (CFS) | The Rockwood Clinical Frailty Scale (CFS) is a researcher assessed scale of clinical frailty with scores ranging from 1-9 where lower scores correspond to increased frailty. We have reported CFS scores of <5 suggestive of frailty.^17^ |
| Montreal Cognitive Assessment (MoCA) | The Montreal Cognitive Assessment (MoCA) is a researcher administered cognitive function questionnaire across 8 domains. Training was provided to site staff using standardised resources supplied online by MoCA TEST Inc.^18^ The assessment was conducted in English with researchers applying their discretion to exclude participants whose command of English was insufficient to complete the test accurately. Total scores range from 0 to 30. We report total MoCA scores of <23 suggestive of at least Mild Cognitive Impairment.^19^ |
| Spirometry and Pulmonary Function Testing | Due to COVID-19 related restrictions on aerosol-generating procedures during the study period, access to spirometry and lung function was limited. Spirometry and Pulmonary function testing were completed as per ERS/ATS recommendations.^20^ Spirometry and Transfer factor values were converted to SI units if not reported as such by sites. Transfer Capacity of the Lung for the uptake of carbon monoxide (TLCO) and carbon monoxide transfer coefficient (KCO) were obtained from the best of two repeat readings. ERS Reference values were used to calculate % predicted values.^21-23^ FEV_1_/FVC <0·7 was used to define airflow obstruction.^24^ % predicted TLCO <80% was considered indicative of impaired gas transfer. |
| BNP / NT-pro BNP | Brain Natriuretic Peptide (BNP) or N-terminal pro B-type Natriuretic Peptide (NT-pro BNP) were collected according to each site’s routine clinically available assay as a biomarker of heart failure. Three sites submitted BNP results with all of the remaining sites submitting NT-pro BNP results. The threshold values used for BNP was ≥ 100 ng/litre ^25^ and for NT-pro BNP ≥ 400ng/litre ^26^ as suggestive of heart failure. |
| Glycated haemoglobin (HbA1c) | Glycated haemoglobin (HbA1c) was collected as a biomarker of current glycaemic control. We have reported HbA1c levels ≥ 6·5% as suggestive of a diagnosis of diabetes.^27^ |
| C-Reactive Protein (CRP) | C-Reactive Protein (CRP) levels were collected as a biomarker of current systemic inflammation. Values reported as below the lower or upper limit reportable range for the assay used at the site have been included at the stated less than or more than cut off value for calculation of mean (SD) results. We have reported CRP levels > 5 mg/L as suggestive of systemic inflammation. |
| **Table 4** |  |
| Symptoms at five-month and one-year visits | Symptom severity was rated using a 0-10 visual analogue scale for Breathlessness, Cough, Fatigue, Sleep quality and Pain before COVID-19 illness and worst in the last 24 hours. |
| EQ-5D-5L VAS | The EQ-5D-5L Visual Analogue Scale is a patient reported outcome questionnaire recording the patient’s self-rated health and was completed for “before your COVID-19 illness” and “your own health state today.” Scores are presented as mean and standard deviation.^28^ |
| EQ-5D-5L Utility Index | The EQ-5D-5L is a five-dimension patient reported outcome questionnaire recording a patient’s self-rated health state for mobility, self-care, usual activities, pain/discomfort and anxiety/depression. These scores are then mapped to a United Kingdom specific Utility Index anchored at 1 for “perfect health” and 0 for “dead” calculated from reported EQ5D-5L scores across the five dimensions.^29^ |
| Washington Group Short Set of Functioning Severity Continuum | The Washington Group Short Set of Functioning (WG-SS) is a patient reported outcome questionnaire using six questions to assess disability and function. Participant responses were transformed to the “Severity Continuum” by assigning scores of zero to responses “no difficulty”, one to responses “some difficulty”, six to responses “a lot of difficulty” and 36 to responses “cannot do at all”. ^30^ |

# Supplementary Data – Results

**Table S1. The participants’ pre-existing comorbidities stratified by tier allocation.**

|  | **Complete PHOSP cohort (n=7935)** | **Tier 1**  **(n=5238)** | **Tier 2**  **(n=2697)** |
| --- | --- | --- | --- |
| **Cardiovascular** |  |  |  |
| Myocardial Infarction | 375 (4.7%) | 259 (4.9%) | 116 (4.3%) |
| Ischaemic Heart Disease | 535 (4.67%) | 370 (7.1%) | 165 (6.1%) |
| Atrial Fibrillation | 473 (5.9%) | 348 (6.6%) | 125 (4.6%) |
| Hypertension | 2946 (37.1%) | 2012 (38.4%) | 934 (34.6%) |
| Congestive Heart Failure | 169 (2.1%) | 132 (2.5%) | 37 (1.4%) |
| Congenital heart disease | 38 (0.5%) | 26 (0.5%) | 12 (0.4%) |
| Valvular heart disease | 115 (1.4%) | 80 (1.5%) | 35 (1.3%) |
| Pacemaker/Implantable defibrillator | 106 (1.3%) | 83 (1.6%) | 23 (0.9%) |
| Peripheral Vascular Disease | 104 (1.3%) | 67 (1.3%) | 37 (1.4%) |
| Hypercholesterolemia/dyslipidaemia | 1,289 (16.2%) | 771 (14.7%) | 518 (19.2%) |
| Cerebrovascular Accident/ Transient  Ischaemic Attack | 304 (3.8%) | 195 (3.7%) | 109 (4.0%) |
| **Neurological and Psychiatric** |  |  |  |
| Dementia | 27 (0.3%) | 22 (0.4%) | 5 (0.2%) |
| Anxiety or Depression | 1367 (17.2%) | 912 (17.4%) | 455 (16.9%) |
| Chronic fatigue syndrome/ fibromyalgia or chronic pain | 339 (4.3%) | 199 (3.8%) | 140 (5.2%) |
| Previous treatment with antidepressants | 921 (11.6%) | 593 (11.3%) | 328 (12.2%) |
| Any previous treatment by a mental health professional | 349 (4.4%) | 191 (3.6%) | 158 (5.9%) |
| **Respiratory** |  |  |  |
| COPD | 602 (7.6%) | 461 (8.8%) | 141 (5.2%) |
| Asthma | 1481 (18.7%) | 999 (19.1%) | 482 (17.9%) |
| Interstitial lung disease | 37 (0.5%) | 28 (0.5%) | 9 (0.3%) |
| Bronchiectasis | 120 (1.5%) | 79 (1.5%) | 41 (1.5%) |
| Obstructive sleep apnoea | 362 (4.6%) | 226 (4.3%) | 136 (5.0%) |
| Obesity hypoventilation syndrome | 21 (0.3%) | 14 (0.3%) | 7 (0.3%) |
| Pleural effusion | 49 (0.6%) | 34 (0.6%) | 15 (0.6%) |
| **Rheumatological** |  |  |  |
| Connective tissue disease | 35 (0.4%) | 23 (0.4%) | 12 (0.4%) |
| Rheumatoid arthritis | 247 (3.1%) | 166 (3.2%) | 81 (3.0%) |
| Osteoarthritis | 813 (10.2%) | 545 (10.4%) | 268 (9.9%) |
| **Gastrointestinal** |  |  |  |
| Peptic ulcer disease | 80 (1.0%) | 49 (0.9%) | 31 (1.1%) |
| Liver Disease - *mild* | 98 (1.2%) | 57 (1.1%) | 41 (1.5%) |
| Liver Disease – *moderate/severe* | 76 (0.9%) | 39 (0.7%) | 37 (1.4%) |
| Gastro-oesophageal reflux disease | 665 (8.4%) | 379 (7.2%) | 286 (10.6%) |
| Inflammatory bowel disease | 116 (1.5%) | 72 (1.4%) | 44 (1.6%) |
| Irritable bowel syndrome | 211 (2.7%) | 132 (2.5%) | 79 (2.9%) |
| **Metabolic/Endocrine/Renal** |  |  |  |
| Type 1 diabetes | 79 (1.0%) | 51 (1.0%) | 28 (1.0%) |
| Type 2 diabetes | 1683 (21.2%) | 1146 (21.9%) | 537 (19.9%) |
| Hypothyroidism | 465 (5.9%) | 322 (6.1%) | 143 (5.3%) |
| Hyperthyroidism | 66 (0.8%) | 41 (0.8%) | 25 (0.9%) |
| Chronic Kidney Disease | 432 (5.4%) | 322 (6.1%) | 110 (4.1%) |
| **Malignancy** |  |  |  |
| Solid Tumour Malignancy - localised | 319 (4.0%) | 227 (4.3%) | 92 (3.4%) |
| Solid Tumour Malignancy - metastatic | 42 (0.5%) | 33 (0.6%) | 9 (0.3%) |
| Leukaemia | 53 (0.7%) | 31 (0.6%) | 22 (0.8%) |
| Lymphoma | 53 (0.7%) | 35 (0.7%) | 18 (0.7%) |
| **Chronic Infectious Diseases** |  |  |  |
| HIV | 22 (0.3%) | 14 (0.3%) | 8 (0.3%) |
| Chronic viral hepatitis | 57 (0.7%) | 30 (0.6%) | 27 (1.0%) |
| Mycobacterium Tuberculosis | 65 (0.8%) | 40 (0.8%) | 25 (0.9%) |

Missing not included in %. COPD=Chronic obstructive pulmonary disease. HIV=Human immunodeficiency virus.

**Table S2: Comparison of the participants’ characteristics between the 1-year visit attendees and Tier 2 participants who failed to return to a 1-year visit.**

|  | **Participants who didn’t attend a 1-year visit (n=597)** | **Participants who attended a 1-year visit (n=2100)** |
| --- | --- | --- |
| Admission at age, years† | 54.6 (13.6) | 58.9 (12.1) |
| Sex |  |  |
| Female | 227 (38.1%) | 811 (38.6%) |
| Male | 369 (61.9%) | 1289 (61.4%) |
| Ethnicity |  |  |
| White | 398 (67.7%) | 1609 (76.9%) |
| South Asian | 100 (17.0%) | 205 (9.8%) |
| Black | 51 (8.7%) | 142 (6.8%) |
| Mixed | 10 (1.7%) | 45 (2.2%) |
| Other | 29 (4.9%) | 89 (4.3%) |
| Missing data | 9 | 10 |
| Index of multiple deprivation score (IMD) |  |  |
| 1 (most deprived) | 152 (25.9%) | 466 (22.3%) |
| 2 | 162 (27.7%) | 460 (22.0%) |
| 3 | 81 (13.8%) | 382 (18.3%) |
| 4 | 101 (17.2%) | 371 (17.7%) |
| 5 (least deprived) | 90 (15.4%) | 412 (19.7%) |
| Missing data | 11 | 9 |
| Body-mass index (BMI) |  |  |
| Median †† | 31.1 [27.3 - 36.2] | 31.2 [27.7- 35.6] |
| <30 kg/m^2^ | 182 (45.0%) | 613 (41.7%) |
| ≥30 kg/m^2^ | 222 (55.0%) | 856 (58.3%) |
| Missing data | 193 | 631 |
| Healthcare worker | 97 (17.6%) | 279 (13.9%) |
| Missing data | 46 | 96 |
| WHO clinical progression scale |  |  |
| WHO class 3-4 | 126 (21.1%) | 321 (15.3%) |
| WHO class 5 | 240 (40.2%) | 895 (42.6%) |
| WHO class 5 | 129 (21.6%) | 504 (24.0%) |
| WHO class 7-9 | 102 (17.1%) | 380 (18.1%) |
| Comorbidities |  |  |
| Median number of comorbidities †† | 1 [0-3] | 2 [1-3] |
| 0 | 172 (28.8%) | 495 (23.6%) |
| 1 | 129 (21.6%) | 442 (21.0%) |
| ≥2 | 296 (49.6%) | 1163 (55.4%) |
| Cardiovascular | 247 (41.4%) | 992 (47.2%) |
| Respiratory | 154 (25.8%) | 570 (27.1%) |
| Type 2 diabetes | 110 (18.5%) | 427 (20.4%) |
| Neuro-psychiatric | 131 (21.9%) | 431 (20.5%) |
| Renal and endocrine | 53 (8.9%) | 234 (11.1%) |
| Admission duration, days† | 12.1 (14.9) | 14.6 (18.7) |
| Positive SARS-CoV-2 PCR | 495 (92.4%) | 1788 (92.6%) |
| Missing data | 61 | 169 |
| Systemic steroids | 293 (51.7%) | 1,155 (57.9%) |
| Missing data | 30 | 106 |
| Antibiotic therapy | 468 (79.9%) | 1,607 (78.5%) |
| Missing data | 11 | 54 |
| Anti-coagulants | 245 (42.7%) | 928 (46.6%) |
| Missing data | 23 | 109 |
| Recovery cluster at 5-month visit | 524 |  |
| Very Severe | 117 (22.3%) | 386 (20.5%) |
| Severe | 134 (25.6%) | 502 (26.7%) |
| Moderate with cognitive impairment | 117 (22.3%) | 426 (22.7%) |
| Mild | 156 (29.8%) | 567 (30.1%) |
| Missing data | 73 | 219 |

Data are n (%) unless † mean (SD) or †† median [IQR]. Percentages are calculated by category after exclusion of missing data for that variable. WHO=World Health Organisation. WHO classes are as follows: 3–4=no continuous supplemental oxygen needed; 5=continuous supplemental oxygen only; 6=continuous or bi-level positive airway pressure ventilation or high-flow nasal oxygen; and 7–9=invasive mechanical ventilation or other organ support. IMD=Index of Multiple Deprivation. BMI=body-mass index. SARS-CoV-2 PCR=severe acute respiratory syndrome coronavirus 2 polymerase chain reaction. See Table S1 for further descriptions of variables.

**Table S3. Total number of received biological research samples.**

|  | **Time points** | | |
| --- | --- | --- | --- |
| **Kit Type** | **5-month** | **1-year** | **Total** |
| Blood | 2,308 | 1,779 | 4,087 |
| Oral rinse | 1,626 | 1,394 | 3,020 |
| Sputum (spontaneous) | 326 | 197 | 523 |
| Urine | 2,085 | 1,706 | 3,791 |
| Saliva (for DNA analysis) * | - | - | 2,638 |

*Saliva samples were collected once from a subset of tier 1 participants only. DNA= Deoxyribonucleic acid. RNA= Ribonucleic acid

**Table S4. Total number of acquired radiological images (X-rays and thoracic CT scans).**

| **Type of imaging** | **Tier 1** | **Tier 2** | **Total** |
| --- | --- | --- | --- |
| Non-contrast thoracic CT scans | 113 | 260 | 373 |
| Contrast thoracic CT scans | 93 | 207 | 300 |
| Chest X-Rays | - | - | 3903 |

CT scans= Computed Tomography Scan.

**Table S5. Total number of acquired MRI scans as per the C-MORE sub-study per contributing sites.**

| **Centre Name** | **N of participants** | **Received Studies** |
| --- | --- | --- |
| University Hospitals of Leicester NHS Trust | 99 | 99 |
| Oxford University Hospitals NHS Foundation Trust | 61 | 61 |
| Sheffield Teaching Hospitals NHS Foundation Trust | 48 | 48 |
| Leeds Teaching Hospitals NHS Trust | 39 | 47 |
| Imperial College Healthcare NHS Trust | 34 | 34 |
| Kings College Hospital NHS Foundation Trust | 33 | 33 |
| University College London Hospitals NHS Foundation Trust | 33 | 45 |
| Manchester University NHS Foundation Trust | 24 | 24 |
| Liverpool University Hospitals NHS Foundation Trust | 20 | 42 |
| Nottingham University Hospitals NHS Trust | 13 | 13 |
| St Bartholomews Hospital, Barts Health NHS Trust | 10 | 11 |
| University Hospitals Birmingham NHS Foundation Trust | 10 | 47 |
| Royal Papworth Hospital | 8 | 7 |
| Total | 432 | 511 |

**Table S6. Final list of top 10 research questions (not ranked).**

| **No** | **Research question** |
| --- | --- |
| 1. | What are the underlying mechanisms of long COVID that drive symptoms and/or organ impairment? |
| 2. | What imaging techniques or scans may be able to detect and predict the development of organ problems or wider systemic issues? |
| 3. | What happens to the immune system throughout patients’ recovery from COVID-19? |
| 4. | What can data at 6 and 12 months tell us about the long-term trajectory of illness? |
| 5. | What blood or other laboratory tests may be able to detect and predict the development of organ problems or wider systemic issues? |
| 6. | What is the impact of treatment(s) during the acute (initial) stage of COVID-19 on recovery? |
| 7. | What are the problems within the muscles associated with symptoms limiting activity/function/exercise? If so, what can be done to help? |
| 8. | What medications, dietary changes, supplements, rehabilitation and therapies aid recovery? |
| 9. | What can be done to support mental well-being during recovery? |
| 10. | What is the risk of future adverse health events (e.g., stroke, heart attack)? |

**Table S7. Change in occupation status COVID stratified by the two research visits.**

|  | **5-month (n=2570)** | | **1-year (n=2100)** | |
| --- | --- | --- | --- | --- |
|  | **n** | **%** | **n** | **%** |
| Working full-time or part-time before COVID-19 | 1137 | 54.2% | 914 | 52.8% |
| Missing data | 471 |  | 369 |  |
| No longer working after COVID-19 | 222 | 11.0% | 121 | 8.5% |
| Missing data | 554 |  | 681 |  |
| Occupation change due to health after COVID-19 | 803 | 46.7% | 326 | 34.6% |
| Missing data | 849 |  | 1159 |  |

# References

1. Office for National Statistics. National Statistics Postcode Lookup (February 2020). February 02, 2020. https://geoportal.statistics.gov.uk/datasets/national-statistics-postcode-lookup-february2020 (01 December 2022, date last accessed).
2. WHO Working Group on the Clinical Characterisation and Management of COVID-19 infection. “A minimal common outcome measure set for COVID-19 clinical research.” *The Lancet. Infectious diseases* 2020; **20**(8): e192-e197.
3. Johnson SU, Ulvenes PG, Øktedalen T, Hoffart A. Psychometric properties of the general anxiety disorder 7-item (GAD-7) scale in a heterogeneous psychiatric sample. *Front Psychol* 2019; **10**:1713.
4. Levis B, Benedetti A, Thombs BD. Accuracy of Patient Health Questionnaire-9 (PHQ-9) for screening to detect major depression: individual participant data meta-analysis. *BMJ* 2019; **365**:l1476.
5. Contractor AA, Elhai JD, Fine TH, et al. Latent profile analyses of posttraumatic stress disorder, depression and generalized anxiety disorder symptoms in trauma-exposed soldiers. *J Psychiatr Res* 2015; **68**: 19-26.
6. Weathers FWL, B.T. Keane, T.M. Palmieri, P.A. Marx, B.P. & Schnurr, P.P. The PTSD Checklist for DSM-5 (PCL-5). 2013. www.ptsd.va.gov (01 March 2021, date last accessed).
7. Yorke J, Moosavi SH, Shuldham C, Jones PW. Quantification of dyspnoea using descriptors: development and initial testing of the Dyspnoea-12. *Thorax* 2010; **65**(1): 21-6.
8. FACIT.org. Functional Assessment of Chronic Illness Therapy – Fatigue: A 13-item FACIT Fatigue Scale. https://www.facit.org/measures/FACIT-F (19 March 2021, date last accessed).
9. Butt Z, Lai JS, Rao D, Heinemann AW, Bill A, Cella D. Measurement of fatigue in cancer, stroke, and HIV using the Functional Assessment of Chronic Illness Therapy - Fatigue (FACIT-F) scale. *J Psychosom Res* 2013; **74**(1): 64-8.
10. Cleeland CS. The Brief Pain Inventory User Guide. 2009. https://www.mdanderson.org/documents/Departments-and-Divisions/SymptomResearch/BPI_UserGuide.pdf (01 March 2021, date last accessed).
11. Cleeland CS, Ryan KM. Pain assessment: global use of the Brief Pain Inventory. *Ann Acad Med Singapore* 1994; **23**(2): 129-38.
12. Vasunilashorn S, Coppin AK, Patel KV, et al. Use of the Short Physical Performance Battery Score to predict loss of ability to walk 400 meters: analysis from the InCHIANTI study. *J Gerontol A Biol Sci Med Sci* 2009; **64**(2): 223-9.
13. Guralnik JM, Simonsick EM, Ferrucci L, et al. A short physical performance battery assessing lower extremity function: association with self-reported disability and prediction of mortality and nursing home admission. *J Gerontol* 1994; **49**(2): M85-M94.
14. Fish J. Short Physical Performance Battery. In: Kreutzer JS, DeLuca J, Caplan B, eds. Encyclopedia of Clinical Neuropsychology. New York, NY: Springer New York; 2011: 2289-91.
15. Singh SJ, Morgan M, Scott S, Walters D, Hardman AE. Development of a shuttle walking test of disability in patients with chronic airways obstruction. *Thorax* 1992; **47**(12): 1019-24.
16. Probst VS, Hernandes NA, Teixeira DC, et al. Reference values for the incremental shuttle walking test. *Respir Med* 2012; **106**(2): 243-8.
17. Rockwood K, Song X, MacKnight C, et al. A global clinical measure of fitness and frailty in elderly people. *CMAJ* 2005; **173**(5): 489-95.
18. Nasreddine ZS. MoCA Montreal Cognitive Assessment Training & Certification. https://www.mocatest.org/training-certification/ (01 March 2021, date last accessed).
19. Carson N, Leach L, Murphy KJ. A re-examination of Montreal Cognitive Assessment (MoCA) cutoff scores. *Int J Geriatr Psychiatry* 2018; **33**(2): 379-88.
20. Graham BL, Steenbruggen I, Miller MR, et al. Standardization of spirometry 2019 update. An official American thoracic Society and European respiratory Society technical statement. *Am J Respir Crit Care Med* 2019; **200**(8): e70-e88.
21. Quanjer PH, Stanojevic S, Cole TJ, et al. Multi-ethnic reference values for spirometry for the 3–95-yr age range: the global lung function 2012 equations. Eur Respiratory Soc; 2012. 40
22. Stanojevic S, Graham BL, Cooper BG, et al. Official ERS technical standards: Global Lung Function Initiative reference values for the carbon monoxide transfer factor for Caucasians (vol 50, 1700010, 2017). *Eur Respir J* 2020; **56**(4).
23. Stanojevic S, Graham BL, Cooper BG, et al. Official ERS technical standards: Global Lung Function Initiative reference values for the carbon monoxide transfer factor for Caucasians. *Eur Respir J* 2017; **50**(3).
24. Vestbo J, Hurd SS, Agustí AG, et al. Global strategy for the diagnosis, management, and prevention of chronic obstructive pulmonary disease: GOLD executive summary. *Am J Respir Crit Care Med* 2013; **187**(4): 347-65.
25. Acute heart failure: diagnosis and management. National Institute for Health and Care Excellence; 2014.
26. Chronic heart failure in adults: diagnosis and management. National Institute for Health and Care Excellence; 2018.
27. International Expert Committee Report on the Role of the A1C Assay in the Diagnosis of Diabetes. *Diabetes Care* 2009; **32**(7): 1327-34.
28. Herdman M, Gudex C, Lloyd A, et al. Development and preliminary testing of the new five level version of EQ-5D (EQ-5D-5L). *Qual Life Res* 2011; **20**(10): 1727-36.
29. Gerlinger C, Bamber L, Leverkus F, et al. Comparing the EQ-5D-5L utility index based on value sets of different countries: impact on the interpretation of clinical study results. *BMC Res Notes* 2019; **12**(1): 1-6.
30. Creating Disability Severity Indicators Using the WG Short Set on Functioning (WG-SS) (CSPro). January 2021. https://www.washingtongroup-disability.com/fileadmin/uploads/wg/WG_Document__5H_-_Analytic_Guidelines_for_the_WG-SS__Severity_Indicators_-_CSPro_.pdf (16 November 2021, date last accessed).
